# Proteins maintain hydration at high [KCl] concentration regardless of content in acidic amino acids

**DOI:** 10.1016/j.bpj.2021.05.015

**Published:** 2021-06-02

**Authors:** Hosein Geraili Daronkola, Ana Vila Verde

**Affiliations:** 1 Department of Theory & Bio-Systems, Max Planck Institute of Colloids and Interfaces, Potsdam, Germany

## Abstract

Proteins of halophilic organisms, which accumulate molar concentrations of KCl in their cytoplasm, have a much higher content in acidic amino acids than proteins of mesophilic organisms. It has been proposed that this excess is necessary to maintain proteins hydrated in an environment with low water activity, either via direct interactions between water and the carboxylate groups of acidic amino acids or via cooperative interactions between acidic amino acids and hydrated cations. Our simulation study of five halophilic proteins and five mesophilic counterparts does not support either possibility. The simulations use the AMBER ff14SB force field with newly optimized Lennard-Jones parameters for the interactions between carboxylate groups and potassium ions. We find that proteins with a larger fraction of acidic amino acids indeed have higher hydration levels, as measured by the concentration of water in their hydration shell and the number of water/protein hydrogen bonds. However, the hydration level of each protein is identical at low (*b*_KCl_ = 0.15 mol/kg) and high (*b*_KCl_ = 2 mol/kg) KCl concentrations; excess acidic amino acids are clearly not necessary to maintain proteins hydrated at high salt concentration. It has also been proposed that cooperative interactions between acidic amino acids in halophilic proteins and hydrated cations stabilize the folded protein structure and would lead to slower dynamics of the solvation shell. We find that the translational dynamics of the solvation shell is barely distinguishable between halophilic and mesophilic proteins; if such a cooperative effect exists, it does not have that entropic signature.

## Significance

Acidic amino acids are known to bind the most water of all amino acids, so it has been proposed that their excess is necessary to retain protein hydration in environments with low water activity. Our results do not support this scenario for the case of concentrated KCl solutions, typical of the cytoplasm of some halophilic organisms; the 10 proteins we investigate keep their hydration level constant under widely different external KCl concentrations despite having very different content in acidic amino acids. The hydrated electrolyte ions integrate the protein solvation shell rather than compete with the protein for available water. Maintaining hydration in the folded state is not the evolutionary driving force behind the excess acidic amino acids observed in halophilic proteins.

## Introduction

Halophilic organisms, unlike most life on Earth, have the uncanny ability to survive at molar external NaCl concentrations. Despite their relative scarcity, halophilic organisms have been found in multiple kingdoms of life: archaea (1), bacteria (2), protozoa, fungi, algae, and multicellular eukaryotes (3,4). To counterbalance the high osmotic stress induced by high external NaCl concentrations, some halophiles accumulate equivalent concentrations of KCl in their cytoplasm (5). At such high salt concentrations, the interactions that dictate the structure and structural stability of proteins differ markedly from those at the much lower salt concentrations found in most organisms.

As a result, many proteins of mesophilic organisms (here, termed mesophilic proteins) are poorly soluble at KCl concentrations typical of the cytoplasm of halophilic organisms (6, 7, 8). In contrast, proteins of halophilic organisms (here, termed halophilic proteins) are structurally stable and functioning at high salt concentrations, but often show lower (or no) stability and activity at KCl concentrations typical of the cytoplasm of mesophilic organisms (5). Their resilience is related to their amino acid composition (3,5,9, 10, 11). Cytoplasmic proteins of halophilic organisms are, on average, longer than their mesophilic counterparts; they also have a higher fraction of random coil structure at the expense of *α*-helix structure (12); they are richer in small+polar and small+apolar amino acids and poorer in large+hydrophobic amino acids; they are also poorer in cationic amino acids, which contain longer alkyl sections in their side chains than the anionic ones. The reduction in the fraction of large+hydrophobic and cationic amino acids and the increase in small+polar and small+apolar is thought to compensate the increased attraction between nonpolar groups as salt concentration increases (5).

Halophilic proteins are also much richer in acidic amino acids (3,13)—mostly located on their surface (9)—than their mesophilic counterparts, leading to substantially negative net protein charges.

The role played by the excess acidic amino acids, however, is not yet well understood. One of the most intuitive explanations is that large surface charge prevents protein aggregation at high salt concentrations. The excess surface charge in halophilic proteins would compensate for charge screening at high salt concentrations and would prevent aggregation (3,5,14, 15, 16). Several results, however, suggest that it is unlikely that charge repulsion is the only–or even main–function of excess acidic amino acids. Experiments show that the stability of halophilic proteins at 0.05 mol/dm^3^ NaCl and ∼$10^{-6}$ mol/dm^3^ NADH^+^ is as high as that reached with molar concentrations of NaCl (17). Charge screening alone does not explain the strong dependence of the stability of the folded state of halophilic proteins, and/or their function, on salt concentrations up to several molar, because measurements (5) and calculations (14) indicate that screening of electrostatic interactions by salt is largely complete at *c*_salt_ = 0.5 mol/dm^3^. At the high salt concentrations seen inside halophiles, electrostatic repulsion between the carboxyl groups at the surface of proteins should have only a small effect on their structural stability, as one NMR study shows (16), and likewise on their ability to aggregate. At present, two other explanations have been proposed for the large fraction of acidic amino acids in halophilic proteins:

### Ion-solvent stabilization model

Experiments have shown that some folded halophilic proteins bind large amounts of water, NaCl, and KCl (18,19); in contrast, salt binding was not detected in mesophilic proteins in the native state (20). Moreover, quasielastic neutron spectroscopy measurements of water translational dynamics in the cytoplasm of halophilic and nonhalophilic bacteria suggest that halophiles have a water fraction with extremely slow dynamics that is absent from nonhalophiles (21). Based on these results, it has been proposed that the abundance of acidic amino acids enables cooperative, stabilizing interactions with cations. The acidic amino acids contribute to a net stabilization of the folded protein structure (despite intramolecular electrostatic repulsion) by forming cooperative hydrated ion networks (20,22). These networks keep the folded protein hydrated despite the high salt concentration and should lead to highly ordered protein hydration shells (3). According to this view, excess acidic amino acids indeed prevents aggregation of proteins at high salt concentration not by charge repulsion but because the cooperative ion-water-protein networks stabilize the protein solvation layer.

### Solvent-only stabilization model

The ion-solvent stabilization model has been challenged, however, based on multiple observations. ^17^O magnetic relaxation experiments did not find any differences in the rotational dynamics in hydration shells of halophilic and nonhalophilic versions of protein L (23), in contrast to the predictions of the ion-solvent stabilization model. Additionally, recent experiments have shown that some extremely halophilic organisms can thrive at low external NaCl concentrations, i.e., also with cytoplasmic KCl concentrations equal to those found in nonhalophiles, despite having a markedly acidic proteome (1); this result suggests that stabilizing interactions between cations and the acidic residues are not critical. To explain these observations, it was proposed that an abundance of acidic amino acids at the surface of halophilic proteins is necessary to compete–rather than cooperate–with the ions in solution for available water and, thus, ensure that the protein surface remains sufficiently hydrated–and the protein remains soluble, conformationally stable, and thus functional–at high salt concentrations (1,24).

Here, we report a molecular dynamics study of five pairs of halophilic and mesophilic proteins in which we comparatively examine how the structure and dynamics of protein hydration shells is affected by the concentration of KCl and by the fraction of acidic amino acids in the protein. We present also parameters for the interaction between K^+^ and carboxylate, critical for this study, optimized to reproduce the experimental solution activity derivative of potassium acetate solutions up to 2 mol/kg and crystallographic information of potassium ions in the vicinity of acidic amino acids.

## Materials and methods

### Force fields

To gain insight into the role played by acidic amino acids in halophilic proteins using simulations, we require force fields for proteins, water, and ions with the correct balance of interactions between all the species, both at low and high (up to several molals) KCl concentrations. The force fields we use—the TIP3P water model (25), the AMBER ff14SB (26,27) force field for proteins, the general AMBER force field (GAFF) for acetate (28), and the potassium and chloride parameters of Joung and Cheatham (29) for TIP3P water meet this requirement for most interactions, so only a few interactions were modified as described below. The AMBER ff14SB force field used with TIP3P water reproduces the hydration free energies of small-molecule analogs of the side chains of neutral amino acids to within 1 kcal/mol root mean-square error of the experimental reference values (30), and the partition coefficients of the same analogs between water and several organic solvents to within 0.5 log units (31). This combination of solvent and protein force field reproduces the secondary structure content of short peptides (27) and the backbone dynamics in the native state of globular proteins (27). The experimental hydration free energies of K^*+*^ and Cl^−^ were one of the target properties used in the parameterization done by Joung and Cheatham (29). Their parameters reproduce the solution activity of aqueous solutions of this salt up to *b* = 2 mol/kg (32), so they are appropriate for both dilute and concentrated solutions. Metallic ligands present in the proteins are simulated using AMBER-compatible parameters reported in the literature. The [2Fe-2S]^2+^ ligand present in both ferredoxin proteins studied here is simulated based on the parameters developed by Carvalho et al. (33) for the [2Fe-2S] ferredoxin from *Mastigocladus laminosus*, with slight modifications: 1) all equilibrium angles are set to the values found in the crystal structure of our ferredoxin proteins. And 2) the force constant for the Fe-S-C angle, missing in the parameter set of Carvalho et al. for the [2Fe-2S]^2+^ from *M. laminosus*, is set to the value reported by the same authors for the desulforedoxin protein from *Desulfovibrio gigas*. The desulforedoxin protein contains Fe(III) coordinated by four cysteines in the same configuration as the ferredoxins studied here. Both carbonic anhydrase proteins studied here have a four-coordinated zinc metal center where the zinc ion is connected to three histidine residues and one water molecule. The metal center is simulated using the zinc AMBER force field (34,35) and the Lennard-Jones (LJ) parameters for the zinc ion are from Li et al. (35).

Modifications to these force fields are indispensable for some of the interactions involving carboxylate groups or K^+^. We modify the self-interaction LJ parameters for all carboxylate oxygens to the values proposed by Kashefolgheta et al. (36), which reproduce the hydration free energy of acetate in TIP3P water better than the original AMBER parameters; note that these parameters are used in all interactions derived using combination rules. The LJ parameters for the interaction between carboxylate and the side chain of the positively charged amino acid lysine are also modified to those in (36) to reduce excessively strong salt bridges. The LJ parameters for the interaction between K^+^ and carboxylate oxygens are modified as described in the following section. Data S1 includes Amber ff14SB, dat, and lib files modified to include the optimized parameters mentioned here. These files can be used to seamlessly generate the final prmtop and inpcrd input files necessary to run simulations with Amber. Example input files for one of the proteins simulated here (protein L) are also given in Data S1.

Results from simulations without any modifications to the default LJ parameters, provided for comparison, are marked as “AMBER and GAFF only.”

### Optimizing parameters for K^+^⋯ carboxylate interactions

Our tests (described in the Results) show that the default parameters for K^+^⋯ carboxylate interactions cause excessive contact between these ions. Because this interaction is critical for our study, our first step was to find optimal parameters to describe it.

Intermolecular interactions between any atoms *i* and *j* have the following form in the AMBER force field:

(1) $$V\left(r_{ij}\right)=\varepsilon_{ij}\left[{\left(\frac{R_{\text{min},ij}}{r_{ij}}\right)}^{12}-2{\left(\frac{R_{\text{min},ij}}{r_{ij}}\right)}^{6}\right]+\frac{q_{i}q_{j}}{4\pi \varepsilon_{0}r_{ij}}$$

In this expression, *q*_*i*_ and *q*_*j*_ are the charges of the atoms, *r*_*ij*_ is the distance between them, *ε*_0_ is the dielectric permittivity in the vacuum. *R*_min,*ij*_ and *ε*_*ij*_ are parameters determining the LJ potential, which mimics the van der Waals interatomic interaction. LJ parameters for the interaction between different atom types are typically obtained from their self-interaction parameters using combination rules:

(2) $$R_{\text{min},ij}=\frac{\left(R_{\text{min},ii}+R_{\text{min},jj}\right)}{2},\varepsilon_{ij}=\sqrt{\left(\varepsilon_{ii}\;\varepsilon_{jj}\right)},$$

where the indices *ii* and *jj* denote the self-interaction parameters.

In the AMBER force field, ions have their nominal charge, and the partial charges of polyatomic ions are determined using a well-defined charge fitting procedure. We opted to tune the K^+^⋯ carboxylate interaction by modifying the LJ potential between K^+^ and the carboxylate oxygen (O) only. The partial charges on the carboxylate atoms remain unchanged, which ensures compatibility within the AMBER force field. In principle, both *R*_min,*ij*_ and *ε*_*ij*_ obtained using Eq. 2 could be overridden by optimized values for the *i* = K^+^, *j* = O pair. This dual optimization is, however, not feasible because of its high computational cost. We optimized only the $R_{\text{min},{\text{K}}^{+}\text{O}}$ parameter and left $\varepsilon_{{\text{K}}^{+}\text{O}}$ at the value obtained with the combination rules. Our prior work confirms that substantial improvements in the description of intermolecular interactions are achieved even with this limited parameterization freedom (36). In Results, we show that the original AMBER parameters lead to substantial deviations between various calculated properties and the experimental reference values. Because of this substantial deviation, the *R*_min_ parameter, with its power of 6 and 12 in the LJ potential, is a better choice for parameterization than *ε*.

The $R_{\text{min},{\text{K}}^{+}\text{O}}$ parameter is optimized to reproduce 1) the experimental solution activity derivative of aqueous solutions of potassium acetate with molality *b*_KCH__^3^COO_ *ϵ*{0.5, 1, 2} mol/kg, and 2) the distances between K^+^ and carboxylate groups found in the crystal structure of the halophilic 2Fe-2S ferredoxin protein with Protein Data Bank, PDB: 1DOI. This protein is one of the few for which the potassium ions present during crystallization were resolved in the crystal structure.

The second target property was added because we found that the solution activity derivative varies nonmonotonically with $R_{\text{min},{\text{K}}^{+}\text{O}}$ to the extent that $R_{\text{min},{\text{K}}^{+}\text{O}}$ values leading to dramatically different solution structure yielded identical solution activity derivatives, as described below. Our final choice of $R_{\text{min},{\text{K}}^{+}\text{O}}$, shown in Results, is that which best reproduces selected distances between K^+^ and carboxylate oxygens in the protein crystal structure while yielding activity derivatives deviating not more than 7% from the reference value for all three concentrations. Our optimized LJ potential for the interactions between K^+^ and carboxylate groups are thus appropriate for simulations of biomolecular systems in a wide range of KCl concentrations and are also appropriate for simulations of systems with acetate ions modeled with the AMBER force field or GAFF.

### Calculating the electrolyte activity derivative in simulations

The molar electrolyte activity derivative can be calculated from simulation using Kirkwood-Buff theory (37,38), which links particle fluctuations to thermodynamic properties. Below, we summarize the main expressions used in this work. Examples of its application to other systems can be found in the literature (39, 40, 41, 42). The central quantity of this theory is the Kirkwood-Buff integral *G*. In this work, we use expressions that enable its use with simulations of closed systems (43). For any two species *i* and *j*, the integral is as follows:

(3) $$G_{ij}\left(R\right)=\int_{0}^{2R}\left[f_{ij}g_{ij}^{NVT}\left(r\right)-1\right]4\pi r^{2}\left(1-3x/2+x^{3}/2\right)dr,$$

Where *R* is 1/4 of the simulation box size, *x* = *r*/(2*R*) and $g_{ij}^{NVT}\left(r\right)$ is the radial distribution function (RDF) in the canonical ensemble. The factor *f*_*ij*_ corrects for the fact that the tail of RDFs in closed systems does not converge to 1. Even though there is no formal relation between the RDFs in open and closed systems, the ratio of their tails is expected to be of the order of 1 ± 1/*N*, where *N* is the number of particles in the simulation box (44, 45, 46). For this reason, a multiplicative correction factor is appropriate.

Because of electroneutrality, applying Kirkwood-Buff theory to an electrolyte solution requires the solution to be treated as a binary system of indistinguishable ions (called the cosolvent) and water (38,40,41). The molar cosolvent activity derivative, *a*_*c*_′, for a solution of a 1:1 electrolyte is thus defined as

(4) $$\begin{array}{c} {{a_{c}}^{\text{′}}=\frac{\partial \ln\;a_{c}}{\partial \ln\;\rho_{c}}|}_{p,T}\\ {=1+\frac{\partial \ln\;\gamma_{c}}{\partial \ln\;\rho_{c}}|}_{p,T} \end{array}$$

In this expression, *γ*_*c*_ is the molar activity coefficient of the cosolvent, *ρ*_*c*_ is its number density, defined as *ρ*_*c*_ = *n*_*c*_/*V*, where *n*_*c*_ = *n*_+_ + *n*___ is the total number of positive and negative ions in the solution volume *V*, and *a*_*c*_ is its molar activity (*a*_*c*_ = *γ*_*c*_ × *ρ*_*c*_). Kirkwood-Buff theory allows the calculation of the electrolyte activity derivative from simulation, as

(5) $${a_{c}}^{\text{′}}=\frac{1}{1+\rho_{c}\left(G_{cc}-G_{cw}\right)}$$

where the subscript *w* refers to the water. The terms in the above expression are sums of Kirkwood-Buff integrals between the ions and water:

(6) $$G_{cc}=\frac{1}{4}\left(2G_{+-}+G_{++}+G_{--}\right)$$

and

(7) $$G_{cw}=G_{+w}+G_{-w}$$

### Experimental electrolyte activity derivative

**Figure 1:**
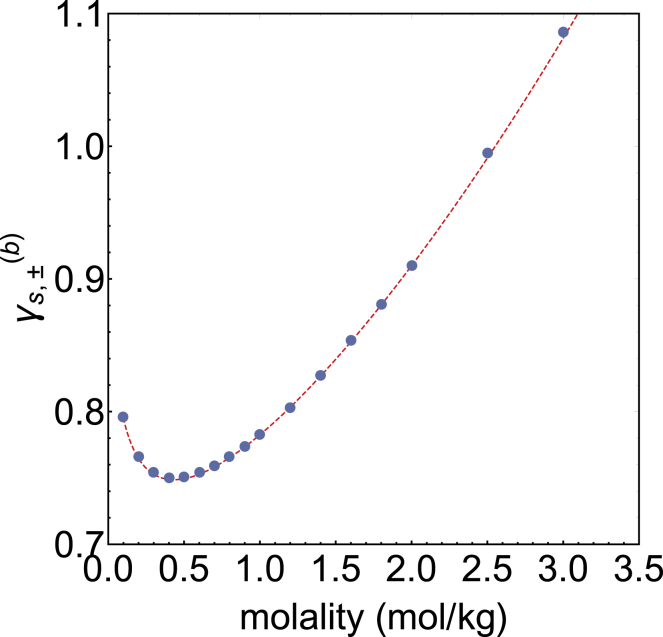
Mean molal activity coefficient $\left(\gamma_{s,\pm }^{\left(b\right)}\right)$ of potassium acetate as a function of the molality of KCH_3_COO. The blue circles are the experimental data (47), and the red dashed line is calculated using the relevant Pitzer equation (48). To see this figure in color, go online.

Experimental activities for electrolytes are typically reported considering the neutral salt unit—as opposed to considering a cosolvent of indistinguishable ions—and in the molal rather than the molar scale. In what follows, the subscript *s* indicates quantities defined in terms of neutral salt units and the superscript (*b*) indicates quantities in the molal scale. The mean molal activity coefficient, $\gamma_{s,\pm }^{\left(b\right)}$, of potassium acetate in aqueous solution has been experimentally determined for molalities up to *b*_*s*_ = 3 mol/kg (47). In Fig. 1, we show the experimental values of $\gamma_{s,\pm }^{\left(b\right)}$ as a function of the molality of KCH_3_COO together with the result of the Pitzer equation applicable to this system (48). The Pitzer equation reproduces the experimental data excellently, so we use it to calculate the molal salt activity derivative

(8) $${a^{'}}_{s}^{\left(b\right)}=\frac{\partial \text{ln}a_{s}^{\left(b\right)}}{\partial \text{ln}b_{s}}{{|}_{p,T}=\frac{\partial \text{ln}\left(\gamma_{s,\pm }^{\left(b\right)}\;b_{s}\right)}{\partial \text{ln}b_{s}}|}_{p,T},$$

using Mathematica.

To enable direct comparisons between experiment and simulation, the experimental molal activity derivative of the salt must first be converted to the corresponding molar quantity:

(9) $${a^{'}}_{s}=\frac{\partial \text{ln}a_{s}}{\partial \text{ln}\rho_{s}}{{|}_{p,T}=1+\frac{\partial \text{ln}\gamma_{s,\pm }}{\partial \text{ln}\rho_{s}}|}_{p,T},$$

where *ρ*_*s*_ is the number density of the salt and *γ*_*s*,±_ its mean molar activity coefficient. The mean molal and molar activity coefficients are related as (49)

(10) $$\gamma_{s,\pm }=\frac{b_{s}\;\rho_{w}}{\rho_{s}}\gamma_{s,\pm }^{\left(b\right)},$$

where *ρ*_*w*_ is the density (mass per unit volume) of water and *ρ*_*s*_ is the number density of the salt, for a 1:1 electrolyte would be *ρ*_*s*_ = *ρ*_*c*_/2. Substituting Eq. 10 into Eq. 9 shows that the salt activity derivatives in the two scales are related as

(11) $${a_{s}}^{{}^{'}}={{a_{s}}^{'}}^{\left(b\right)}\frac{\partial \text{ln}b_{s}}{\partial \text{ln}\rho_{s}}$$

We use Eq. 11 with the solution density reported in (50) to obtain *a*_*s*_′ from *a*_*s*_′^(*b*)^. The molar solution activities expressed in terms of the salt and the cosolvent are related as *a*_*s*_ = 0.5*a*_*c*_ for 1:1 electrolytes; their derivatives and their activity coefficients are identical (*a*_*s*_′ = *a*_*c*_′ and *γ*_*s*,±_ = *γ*_*c*_); the experimental *a*_*s*_′ can thus be directly compared with *a*_*c*_′ calculated from the simulation.

### Simulation details

#### Potassium acetate solutions

We perform molecular dynamics simulations using the GROMACS simulation package in its 2018 version (51,52). Simulations are performed for three different concentrations, *b*_KCH__^3^COO_ *ϵ*{0.5, 1, 2} mol/kg. Each simulation–minimization, equilibration, and production run–is performed for a given test value of $R_{\text{min},{\text{K}}^{+}\text{O}}$, and multiple values are tested at each concentration. These test values are related to the default, $R_{\text{min},{\text{K}}^{+}\text{O},\text{LB}}$ (obtained using the Lorentz-Berthelot combination rule; Eq. 2), via a scaling factor:

(12) $$R_{\text{min},{\text{K}}^{+}\text{O}}=f_{R_{\text{min},{\text{K}}^{+}\text{O}}}\;.\;R_{\text{min},{\text{K}}^{+}\text{O},\text{LB}}$$

For convenience, we refer to the tested values of $R_{\text{min},{\text{K}}^{+}\text{O}}$ via the scaling factor $f_{R_{\text{min},{\text{K}}^{+}\text{O}}}$.

The simulation boxes are prepared by putting 72, 144, or 288 neutral salt units, KCH_3_COO–corresponding to the low, intermediate, and high salt concentrations–in a cubic box with edge length *L* ≈ 6 nm and solvating the systems by adding ∼8000 TIP3P water molecules using the editconf and solvate tools from Gromacs 2018 (51,52). The LJ interactions are cutoff at 1.2 nm. Electrostatic interactions are calculated using direct summation up to 1.2 nm. Beyond this cutoff distance, the electrostatic interactions are calculated with the particle mesh Ewald scheme with a grid spacing of 0.12 nm and sixth order interpolation (53). LJ interactions are smoothly shifted to 0 between 1.0 and 1.2 nm using the switch function available in GROMACS. Long-range dispersion corrections are applied to both the energy and pressure. A leap-frog stochastic (SD) integrator (54) is used to integrate the equations of motion in all simulations, with the temperature fixed at 298 K. All bonds with H-atoms are restrained using the LINCS algorithm (55) in all simulation steps (except the minimization step with l-bfgs method), which enables integration using a 2-fs time step. The systems are equilibrated following a protocol commonly used in biological studies: 1) two initial minimization steps with the steepest-descent and l-bfgs algorithms. The latter is a quasi-Newtonian algorithm for energy minimization which converges faster than the conjugate gradient algorithm. 2) A 500-ps heating equilibration simulation in the canonical ensemble (*NVT*) using Langevin thermostat with a coupling constant of 1.0 ps to 298 K. 3) Another 10-ns simulation is performed in the isothermal-isobaric ensemble (*NpT*) to equilibrate the system density at the pressure of 1 bar, using the Berendsen barostate (56) with a relaxation time of 1.0 ps. We select three distinct configurations from the *NpT* equilibration simulation, with a box volume similar to the average box volume obtained during that simulation. Each of these configurations is then used as the initial state of a production simulation in the *NVT* ensemble and lasting 50 ns, making up 150 ns for each concentration. This large simulation time is necessary to calculate activity derivatives with high precision. The trajectories of production simulations are saved every 2 ps for analysis.

#### Restrained ferredoxin in *c*_KCl_ = 1 mol/dm^3^

Simulations are performed with Gromacs 2018; unless otherwise noted, all simulation details are identical to those used to simulate potassium acetate solutions. Each simulation–minimization, equilibration, and production run–is performed for a given test value of $R_{\text{min},{\text{K}}^{+}\text{O}}$. The simulation box is prepared by solvating the protein with a cubic box of TIP3P water, with the distance between the protein surface and the box face being at least 15 Å. During all simulation steps, the backbone atoms are restrained to their position in the crystal structure at all times, using a harmonic potential with a force constant of 1000 kJ⋅mol^−1^⋅nm^−2^. Applying this restraint is necessary to compare the distance of the K^+^ ions to carboxylate groups between the simulated system and the crystal structure for each value of $R_{\text{min},{\text{K}}^{+}\text{O}}$ tested. A single *NpT* production simulation with duration 200 ns is performed for each $R_{\text{min},{\text{K}}^{+}\text{O}}$.

#### Unrestrained proteins at high and low KCl concentrations

Most simulation steps are performed using the GPU version of the pmemd engine in AMBER 2018 (57). The only exception is the l-bfgs minimization step, which is performed on the Sander engine of the AMBER simulation package because it is not available in the pmemd engine.

The starting configurations for the simulations are prepared using the tLeap module of the AMBER software (58). Each protein is simulated at two different salt concentrations: *b*_KCl_=0.15 mol/kg, corresponding to mesophilic conditions, and *b*_KCl_=2 mol/kg, corresponding to halophilic conditions. The simulation boxes are cubic, with edge length ≈100 Å. Each initial configuration is prepared by putting potassium and chloride ions in numbers corresponding to the desired concentration, and adding extra potassium or chloride for neutralization using the addIons tool of AMBER; the systems are then solvated with the appropriate number of TIP3P water molecules (25) using the tLeap tool from AMBER. The distance between the protein surface and the box face is at least 20 Å in all cases. Periodic boundary conditions are applied in the *XYZ* directions. The nonbonded potential cutoff distance is 12 Å for both LJ and electrostatic interactions. Beyond this cutoff distance, electrostatic interactions are calculated with the particle mesh Ewald scheme with a grid spacing of 1.0 Å, and fourth order interpolation (53). Long-range dispersion corrections are applied to both the energy and pressure. All bonds with H-atoms are constrained using the SHAKE algorithm (59) during the *NpT* equilibration and the production simulations. We use a 2-fs time step for all simulations except for those of the carbonic anhydrase proteins, which use a 1-fs time step. The carbonic anhydrase proteins have a zinc metal center that coordinates with one water molecule; for technical reasons, the SHAKE algorithm cannot be applied to this molecule, which limits the maximal time step that can be used.

**Figure 2:**
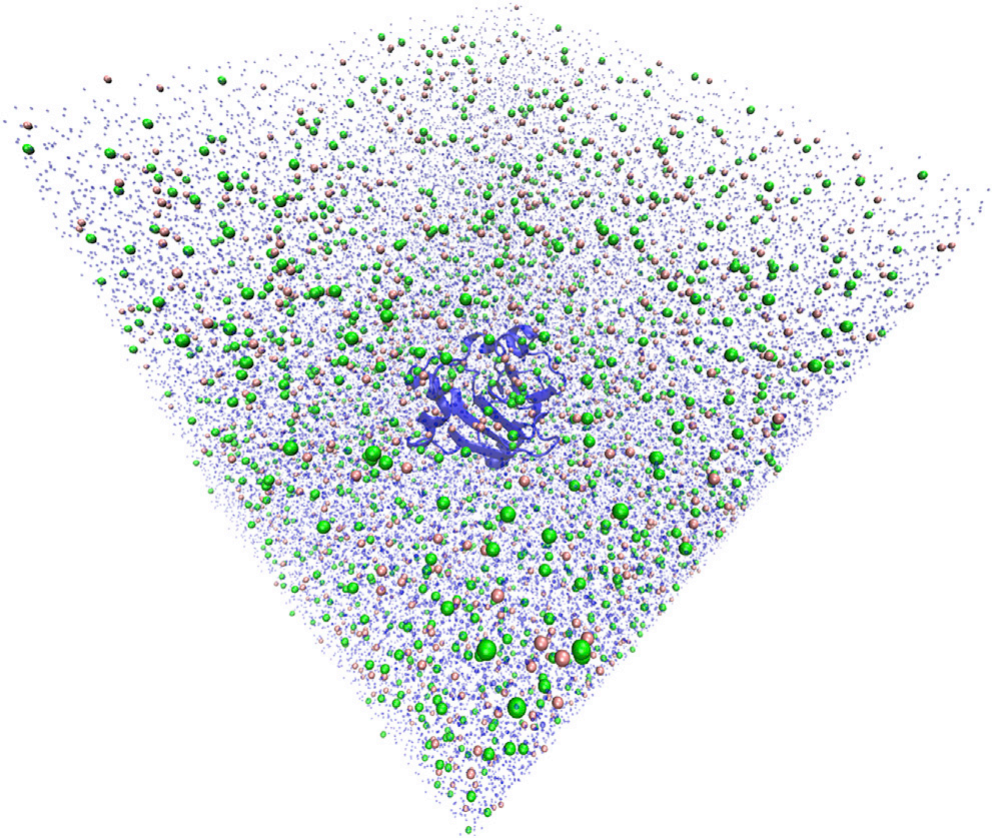
Example simulation box, of the halophilic ferredoxin protein (PDB: 1DOI). Dark blue shape represents the New Cartoon representation of protein, pink spheres represent K^*+*^, green spheres represent Cl^−^, and transparent small blue dots represent water molecules. To see this figure in color, go online.

Each system is subject initially to four minimization cycles, each comprising 2500 minimization steps using the steepest-descent algorithm and 7500 steps using the conjugate gradient algorithm. The protein atoms are restrained to their positions in the pdb file, with the value of the restraint bond constants (500, 300, 100, and 50 kcal⋅mol^−1^⋅Å^−2^) decreasing for each cycle. This procedure removes bad contacts that may be created in the process of adding ions and water to the system. Subsequently, each system is minimized for 10,000 steps using the l-bfgs algorithm without any restraints. The system temperature is progressively increased from 0 to 298 K during a 500-ps simulation in the canonical ensemble (*NVT*), with the protein atoms restrained to their positions with a constant of 10 kcal⋅mol^−1^⋅Å^−2^, using the Langevin thermostat with a collision frequency of 1.0 ps^−1^. Each system is equilibrated for 10 ns in 10 steps of 1 ns in the isothermal-isobaric ensemble (*NpT*) using the Berendsen barostat (56) with a relaxation time of 2.0 ps while increasing the skinnb parameter to 5 Å to prevent unnecessary rebuilds of the pair list when there is no possibility of missing atoms in the pairwise calculation within the cutoff. Increasing the value of this parameter is indispensable because the GPU version of the pmemd engine is very sensitive to changes in the box size. Fig. 2 shows an equilibrated simulation box.

The starting configuration for each production simulation is selected from the last 1 ns of the *NpT* equilibration, under the condition that it has the box density closest to the average for that simulation. The production simulations are performed in the *NVT* ensemble, for 5 × 10^8^ steps, corresponding to 0.5 *μ*s for carbonic anhydrase and 1 *μ*s for all other proteins. The simulations for the carbonic anhydrases were not extended to 1 *μ*s because the proteins remain conformationally stable within the simulated time. For the production simulations, we use the Langevin thermostat with a collision frequency of 0.01 ps^−1^. This low collision frequency is necessary to reduce the impact of the thermostat on the dynamics of the system. Prior work (60) has shown that this low collision frequency leads to self-diffusivity, rotational correlation time, and shear viscosity values within 2% of those obtained from simulations in the *NVE* ensemble at the same temperature. The average temperature in our simulations remains at its target value despite the low collision frequency of the thermostat. Production simulations are saved every 100 ps for analysis.

For the calculation of the mean-square displacement (MSD) for timescales <100 ps, we perform three production simulations for each protein and each KCl concentration, each lasting 1 ns and, with configurations, saved every 100 fs. The starting configuration for each simulation is taken from the longer production runs, at *t ϵ*{100, 500, 900} ns.

## Results

### Optimizing parameters for K^*+*^⋯ carboxylate interactions

**Figure 3:**
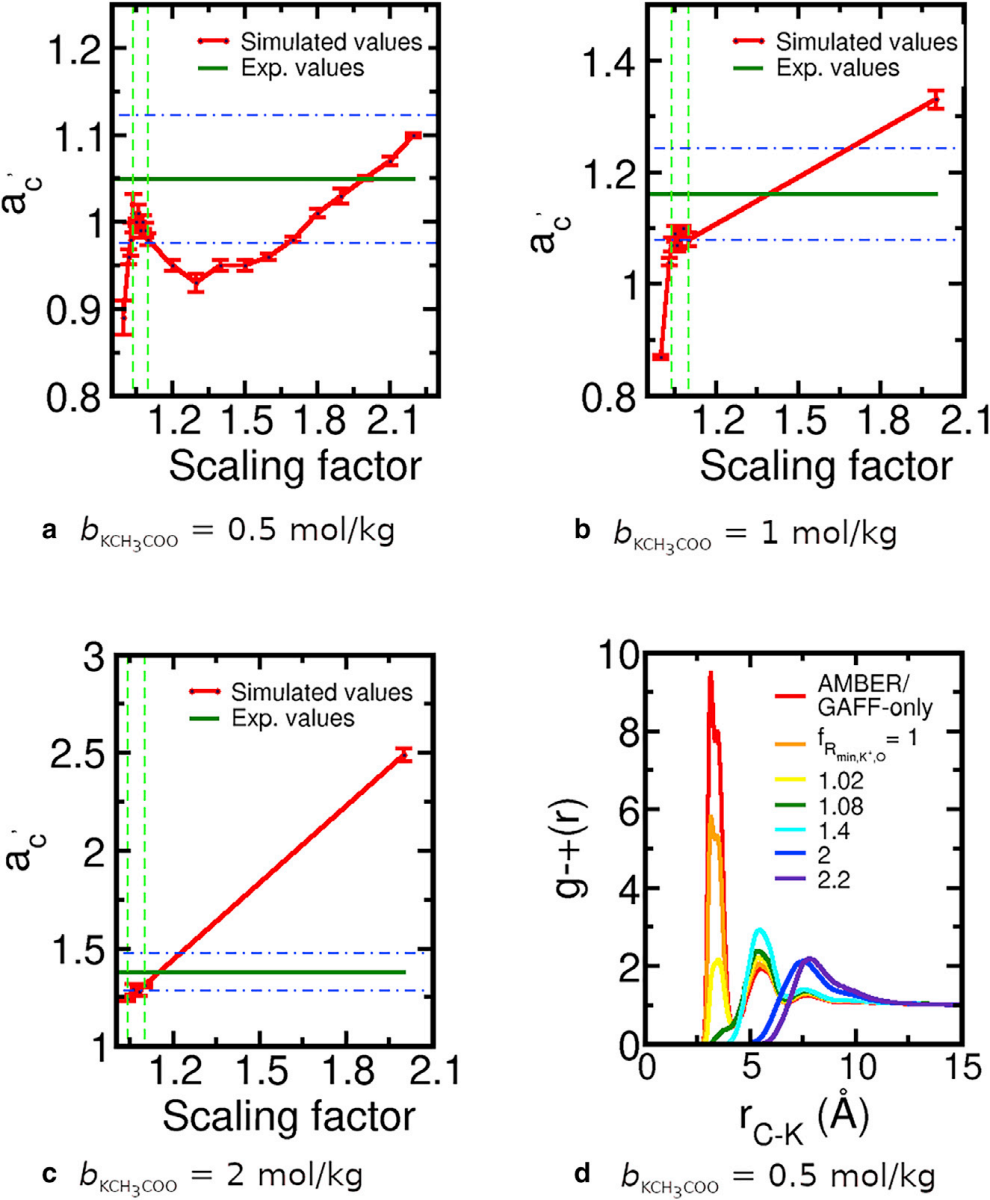
(*a*–*c*) Molar solution activity derivative (*red points*) of potassium acetate solutions as a function of the multiplicative scaling factor ($f_{R_{\text{min},{\text{K}}^{+}\text{O}}}$; see Eq. 12) applied to the LJ $R_{\text{min},{\text{K}}^{+}\text{O},\text{LB}}$ parameter governing the interactions between K^*+*^ and carboxylates. The error bars are the standard error of the mean calculated from three independent production simulations. The red lines are a guide to the eye. The green line shows the experimental reference value; see also Table 2. The area between the two horizontal dashed lines regions show the ±7% deviation from the experimental value. The two vertical dashed lines delimit the range of scaling factors acceptable for the three concentrations. (*d*) RDF of potassium and the carbon bonded to the oxygens of acetate (the same simulations as in *a*) for the indicated values of the scaling factor $f_{R_{\text{min},{\text{K}}^{+}\text{O}}}$. To see this figure in color, go online.

The activity derivative of potassium acetate solutions with molality *b*_KCH__^3^COO_ *ϵ*{0.5, 1, 2} mol/kg calculated in simulation is shown in Fig. 3.

For each concentration, the activity derivative is calculated for different values of $R_{\text{min},{\text{K}}^{+}\text{O}}$, which govern the LJ interaction between K^*+*^ and the carboxylate oxygens; these values are expressed in the figures in terms of a multiplicative scaling factor (defined in Eq. 12). The unoptimized anion-cation parameters, corresponding to the scaling factor of 1 in Fig. 3, yield activity derivatives far from the reference value from experiment (*green lines*). This deviation demonstrates that the unoptimized parameters fail dramatically to adequately describe K^+^⋯ CH_3_COO^−^ interactions in the concentration range we tested; optimizing this interaction is indispensable. With this in mind, we first examine the solution activity derivative for $b_{{\text{KCH}}_{3}\text{COO}}=0.5$ mol/kg (Fig. 3 *a*). It varies nonmonotonically with an increasing scaling factor: initially it rapidly increases, then decreases slowly before again increasing. Perfect agreement with the reference experimental value at this concentration occurs for $f_{R_{\text{min},{\text{K}}^{+}\text{O}}}=2.0$. Despite this agreement, $f_{R_{\text{min},{\text{K}}^{+}\text{O}}}=2.0$ does not result in the correct solution structure at $b_{{\text{KCH}}_{3}\text{COO}}=0.5$ mol/kg: the corresponding anion-cation RDF, shown in Fig. 3 *d*, would suggest that only solvent-separated ion pairs exist in potassium acetate solutions and that neither contact-shared (CIP) nor solvent-shared (SIP) occur (CIP, the two ions are in direct contact; SIP, the ions share one hydration layer; and solvent-separated, each ion retains its first hydration layer). The absence of CIPs disagrees with potentiometric measurements, which indicate that K^+^ and CH_3_COO^−^ associate–albeit weakly–to form neutral complexes (61,62) best understood as pairs of ions in direct contact (63).

The anion-cation RDFs in Fig. 3 *d* indicate that scaling factors in the range $1.02<f_{R_{\text{min},{\text{K}}^{+}\text{O}}}<1.1$ allow the presence of CIPs in solution, i.e., yield a solution structure qualitatively in line with experiment. This range of scaling factors also yields solution activity derivatives within 7% of the target experimental values for all three concentrations (Fig. 3, *a*–*c*). However, despite the fact that solution activity derivatives remain constant for this range of $f_{R_{\text{min},{\text{K}}^{+}\text{O}}}$-values, the solution structure changes substantially: e.g., Fig. 3 *d* shows that for $f_{R_{\text{min},{\text{K}}^{+}\text{O}}}=1.02$, CIPs are still abundant, although much less so than SIPs or 2SIPS; for $f_{R_{\text{min},{\text{K}}^{+}\text{O}}}=1.1$, CIPs are residual. Clearly, randomly choosing a value of $f_{R_{\text{min},{\text{K}}^{+}\text{O}}}$ within the $1.02<f_{R_{\text{min},{\text{K}}^{+}\text{O}}}<1.1$ range is insufficient, and another experimental reference property is desirable to pinpoint the optimal parameter value.

The pdb structure with pdb code 1DOI, of a halophilic ferredoxin, contains five cocrystalized K^+^ near acidic amino acids at the protein surface. This crystal structure was obtained from both room temperature and 100 K difraction data and contains 237 water molecules, corresponding to 55% of the crystal volume. Flash freezing, typically used in crystallography to reach cryogenic temperatures, leaves the solvent in an amorphous state but often contracts the unit cell by 2–7% with most of the contraction arising from the solvent (64). These conditions mean that the protein surface and the potassium ions in this crystal structure are heavily solvated and that the distances between K^+^ and the nearby carboxylates in the crystal structure are a reasonable estimate of the distance between these ions when forming CIPs for the solvated protein at room temperature. We used this information to select the optimal parameter within the $1.02<f_{R_{\text{min},{\text{K}}^{+}\text{O}}}<1.1$ range. To the best of our knowledge, other experimental observables that directly report on the structure of K^+^⋯ carboxylate ion pairs in solvated conditions do not exist. We simulated the ferredoxin protein in *c*_KCl_ = 1 mol/dm^3^ at *T* = 298 K. This high concentration of K^+^ is within the range used in the previous parameterization step. The concentration of ions does not affect the position of the peaks of the RDF, as shown in Fig. 3 *d*, so other concentrations could also have been used; a high concentration is advantageous because it yields RDFs with less noise, given the same simulation time. The backbone atoms were constrained to the coordinates of the 1DOI crystal structure so that any differences in the K^+^⋯ carboxylate interaction between simulations with different $f_{R_{\text{min},{\text{K}}^{+}\text{O}}}$-values reflect the impact of the simulation parameters and not steric changes arising from changes in protein conformation.

**Figure 4:**
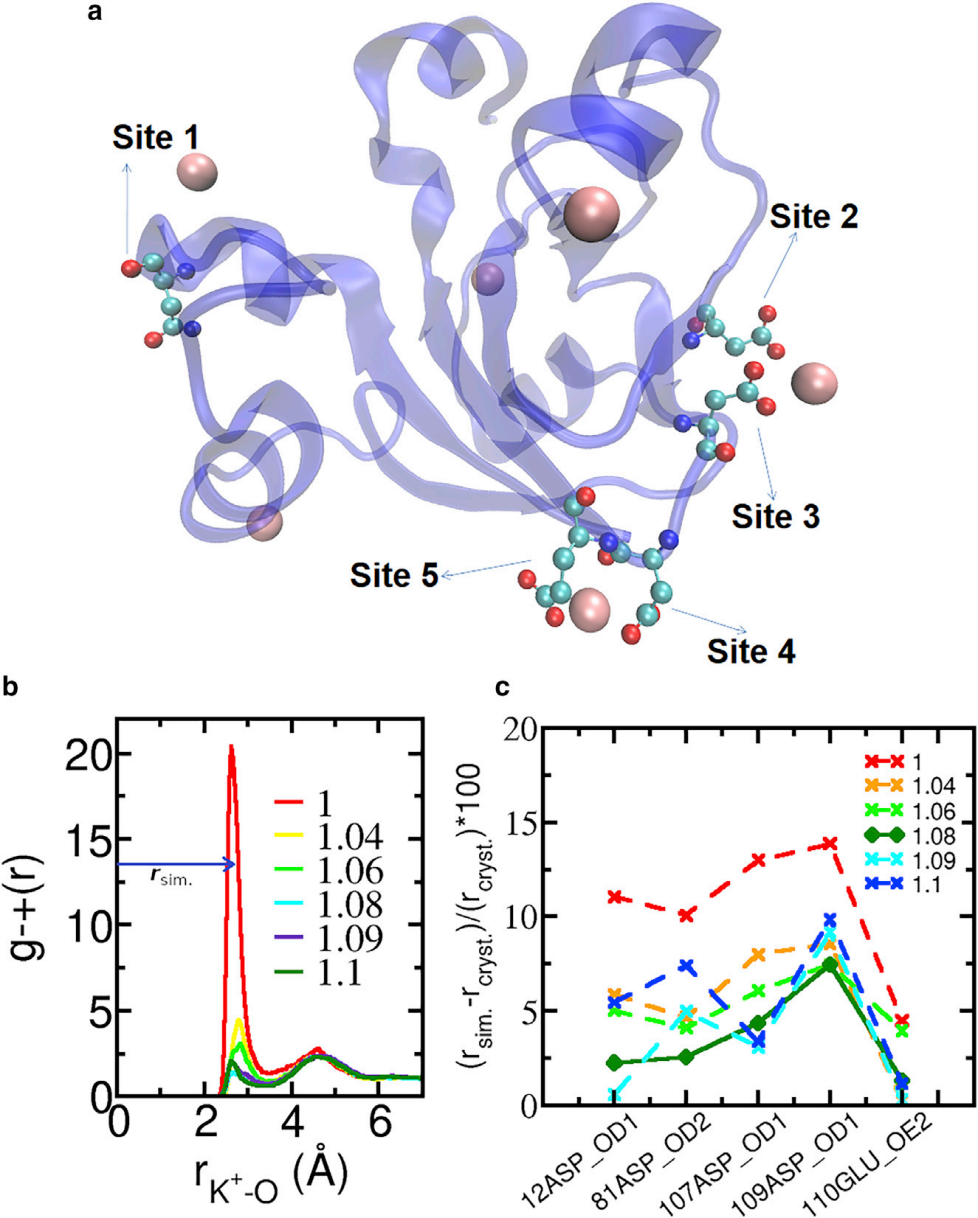
(*a*) Crystal structure (PDB: 1DOI) of the halophilic 2Fe-2S ferredoxin from *Haloarcula marismortui* (24); the K^*+*^ ions are shown in pink. The five acidic amino acid sites that have nearby K^+^, used to parameterize $R_{\text{min},{\text{K}}^{+}\text{O}}$, are indicated. (*b*) Example RDF of K^+^ and the carboxylate oxygens of site 2 at *T* = 298 K and *c*_KCl_ = 1 mol/dm^3^. The distance to the first maximum is identified as r_sim._. The legend shows the values of the scaling factor $f_{R_{\text{min},{\text{K}}^{+}\text{O}}}$. (*c*) Unsigned relative deviation between *r*_sim_ and *r*_cryst_ for the five indicated sites of the halophilic ferredoxin. The protein site is identified by the residue number, residue name, and oxygen name. The legend shows the values of the scaling factor $f_{R_{\text{min},{\text{K}}^{+}\text{O}}}$. Numerical data are shown in Table S1. To see this figure in color, go online.

For each of the five acidic amino acids functioning as the contact site with K^*+*^ in the crystal structure (Fig. 4 *a*), we calculated the carboxylate(O)-K^*+*^ RDF.

Fig. 4*b* shows example RDFs for one of the protein sites and for different values of $f_{R_{\text{min},{\text{K}}^{+}\text{O}}}$. The position, *r*_sim_, of the maximum of the first peak of each RDF was obtained as the mean of a Gaussian curve fitted to each peak. This fit allows a better identification of the position of the peak than taking the data point corresponding to the global maximum of each curve. The *r*_sim_ distances and the corresponding reference values (*r*_cryst_) from the crystal are tabulated in Table S1 for each of the protein sites; *r*_cryst_ is the distance between K^+^ and the carboxylate oxygen identified in Fig. 4 *c*. The *r*_sim_ are always smaller than the reference values for all parameter values tested. The optimal value of $f_{R_{\text{min},{\text{K}}^{+}\text{O}}}$ is selected as that which leads to the smallest deviation between *r*_sim_ and *r*_cryst_. In Fig. 4 *c*, we show the unsigned relative deviation between *r*_sim_ and the reference value from the crystal structure *r*_cryst_ for the five protein sites for multiple values of $f_{R_{\text{min},{\text{K}}^{+}\text{O}}}$. A scaling factor $f_{R_{\text{min},{\text{K}}^{+}\text{O}}}=1.08$ (corresponding to $R_{\text{min},{\text{K}}^{+}\text{O}}^{†}$; Table 1) yields the lowest deviation over all protein sites. Table 2 shows the activity derivative of potassium acetate solutions at the three concentrations investigated, for $f_{R_{\text{min},{\text{K}}^{+}\text{O}}}=1.08$. These values deviate 4.7, 5.3, and 7.2% from the corresponding experimental values for *b*_KCH__^3^COO_ = (0.5, 1, and 2) mol/kg, respectively. The position of the contact ion pair peak between the carboxylate oxygens and K^+^ obtained with the optimal parameter is *r*_sim_ ≈ 2.8 Å, consistent with ab initio calculations using a polarizable continuum model for the solvent (65,66) but larger than the optimal distance of 2.5 Å observed in gas phase calculations (67). Despite the difference in optimal distance observed between those ab initio calculations, both predict that the contact ion pair between carboxylate and K^*+*^ is less stable than that with Na^+^, i.e., these cations follow the normal Hofmeister series when interacting with the carboxylate group. Those results agree with x-ray absorption measurements of acetate solutions (66), with prior molecular simulations using parameters optimized for lower concentrations (68) and with our simulations using our optimized parameters (Fig. S7).

**Table 1:** Recommended value of *R*_min_ for the LJ potential between K^+^ and any carboxylate oxygen when using TIP3P water, the Joung and Cheatham (29) parameters for K^+^, and the GAFF (28) or AMBER (27) force field with the optimized self-interaction parameters for carboxylates from **(**36**)**

Parameter	Source	Value
$R_{\text{min},{\text{K}}^{+}\text{O}}$ (Å)^a^	this work	3.6355
$f_{R_{\text{min},{\text{K}}^{+}\text{O}}}$^b^	this work	1.08
$R_{\text{min},{\text{K}}^{+}\text{O},\text{LB}}$ (Å)^c^	AMBER or GAFF	3.3662

a Given is the optimized parameter value.

b Given is the optimized scaling factor, defined in Eq. 12.

c Given from combination rules (see Eq. 2), provided for comparison.

**Table 2:** Molal and molar solution activity derivative of aqueous solutions of KCH_3_COO with the indicated molality

*b*_KCH__3COO_ (mol/kg)	${a^{'}}_{s}^{\left(b\right)\;\text{exp}}$^a^	${a^{'}}_{s}$^b^	${a^{'}}_{c}$^c^	${a^{'}}_{c}$^d^
0.5	1.0143	1.0488	1.00 ± 0.01	0.83 ± 0.01
1	1.1229	1.1611	1.100 ± 0.004	NC
2	1.3345	1.3798	1.28 ± 0.02	NC

NC, not calculated.

a Given is the experimental molal activity derivative from Pitzer equations (48).

b Given is the experimental molar activity derivative from Pitzer equations and Eq. 11.

c Given is the simulation (this work) of the molar activity derivative using the optimized LJ parameters listed in Table 1 and the self-interaction parameters for carboxylate from (36). The uncertainty is the standard error of the mean, calculated from four independent simulations.

d Given is the simulation (this work) of the molar activity derivative using the AMBER force field or GAFF.

### Structure and dynamics of the hydration shell of mesophilic versus halophilic proteins

The ion-solvent stabilization model and the solvent-only stabilization model, proposed to explain the function of excess acidic amino acids in halophilic proteins, imply that the structure and dynamics of the hydration shell of halophilic proteins differs from that of mesophilic ones: according to both models, excess acidic amino acids are indispensable in halophilic proteins, so they remain hydrated at high KCl concentrations. The solvent-only stabilization model proposes that acidic amino acids enhance hydration by direct interactions with the water and that ion-protein interactions are not relevant for hydration or for protein stability. In contrast, the ion-solvent stabilization model proposes that protein hydration is maintained, and the folded protein structure is stabilized, by cooperative interactions between the acidic amino acids, the cations in solution (K^+^) and water. According to this model, these cooperative interactions are possible only in the folded conformation of halophilic protein because only specific tertiary and quaternary protein structures enable them. These cooperative interactions are responsible for the large amounts of cations bound to halophilic proteins and should result in excessively slow dynamics of water of hydration around those proteins. In what follows, we compare the structure and dynamics of the hydration shells of five pairs of halophilic-mesophilic proteins and discuss the results in the context of the predictions and assumptions of both models.

### Selection of halophilic-mesophilic protein pairs

The five protein pairs investigated here were selected considering the following: 1) availability of experimentally determined structures, 2) existence of experimental studies on their conformational changes and/or activity as a function of salt concentration, 3) availability of parameters for simulation, 4) diversity of size and surface charge density, and 5) similarity of the amino acid sequence and structure between each halophilic protein and its mesophilic pair. In Table S2, we list the pdb IDs of the proteins selected for the study and show the structural and sequence similarity between each of the five halophilic-mesophilic pairs. The pair sequence identity varies between 19 and 90%. Despite this broad range in sequence identity, the proteins in each pair share a common structure, visible in the narrow range of the root mean-square deviation (RMSD) of the C^*α*^ atoms of the backbone of the amino acids between protein pairs, after structural alignment: 0.95 < RMSD/Å < 2.41.

A comparison of four sets of orthologous proteins–homologous protein sequences that share the same ancestral sequence separated by a speciation event–shows that halophilic proteins have typically 17–20% acidic amino acids and only 8–10% basic amino acids, whereas their orthologous mesophilic proteins are weakly negatively charged, having 12–14% acidic and 10–12% basic amino acids. Table S3 shows the length of each sequence, the number of acidic and basic amino acids, and the total charge of the proteins investigated here; these data show that the sets of halophilic and mesophilic proteins are representative of their respective category. The halophilic proteins range from the very highly charged ferredoxin (−29 *e*), in which 27% of amino acids are acidic and only 5% of amino acids are basic, to dihydrofolate reductase (−15 *e*), with only 19 and 9% of acidic and basic amino acids, respectively. The mesophilic proteins have an acidic amino acid content ranging from 8 (*β*-lactamase) to 18% (ferredoxin) and are on average only weakly negative. Both ferredoxin proteins are outliers: the halophilic ferredoxin is the most highly charged protein known, and even the mesophilic ferredoxin has an unusually high negative charge.

### Structural stability

We simulate each protein at both low (*b*_KCl_ = 0.15 mol/kg) and high (*b*_KCl_ = 2 mol/kg) concentration of KCl. The mesophilic proteins at high salt concentration and the halophilic ones at low salt concentration are thus under nonnatural conditions. Experiments have shown that the structure of proteins is typically more stable, and proteins have higher activity when in media with their natural KCl concentration (5). Mesophilic proteins are expected to have low solubility at high KCl or NaCl concentrations; under those conditions, many mesophilic proteins have a negative osmotic second virial coefficient (6,7), known to correlate strongly with low protein solubility (8).

Within the duration of the production simulations—0.5 *μ*s for the carbonic anhydrase proteins and 1 *μ*s for the others—all proteins retain their structure irrespective of the concentration of KCl. Comparison with experimental data suggests that under non-natural electrolyte conditions, the native fold of some of the proteins studied here indeed continues to be the stable state. This seems to be the case for the halophilic and mesophilic carbonic anhydrases (12) and the halophilic *β*-lactamase (69) studied here: experimental measurements of protein activity indicate that these proteins remain active–and thus presumably folded and in soluble form–under non-natural KCl concentrations. For other proteins, however, the conformational stability seen in the simulation may reflect a metastable state with lifetime longer than the simulation time. This may be the case for the halophilic protein L: experimental studies show that it is largely unfolded at low salt concentrations (23), even though it remains folded during the simulation.

### Solvation layer structure

**Figure 5:**
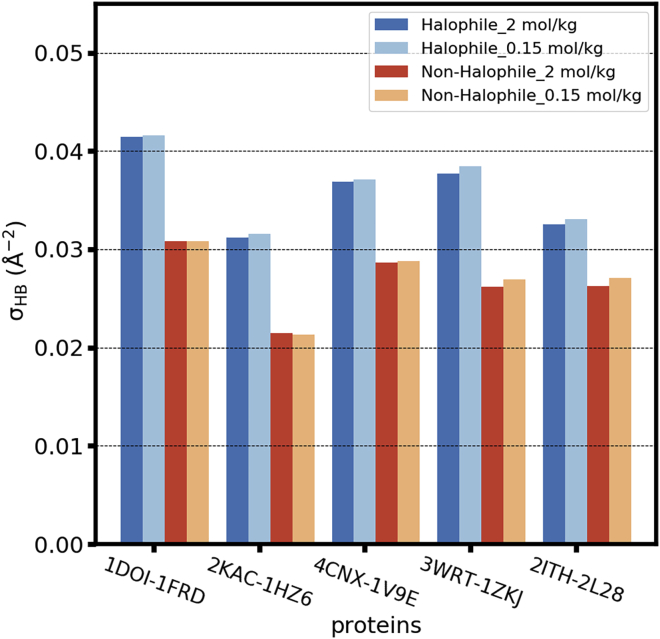
Surface density of water-protein hydrogen bonds for the indicated halophilic-mesophilic proteins, identified by their pdb ID, for different KCl concentrations. To see this figure in color, go online.

We first assess how hydrogen bonds donated by water molecules to the protein (termed water-protein hydrogen bonds) are affected by salt concentration. We focus our attention on strong hydrogen bonds (70), present if the distance between the water oxygen and the hydrogen bond acceptor (N, O) is below 3 Å, and the angle formed by the water hydroxy, and the acceptor is larger than 135°. In Fig. 5, we show the surface density, *σ*_HB_, of water-protein hydrogen bonds, calculated as

(13) $$\sigma_{\text{HB}}=\frac{\bar{n_{\text{HB}}}}{\bar{SASA}},$$

where $\bar{n_{\text{HB}}}$ is the time-averaged total number of water-protein hydrogen bonds and $\bar{SASA}$ is the time-averaged solvent-accessible surface area of each protein at the position of the first maximum of the hydration layer of each protein. Further details about the SASA calculation are provided below in the text accompanying Eq. 14.

#### Surface density of water-protein hydrogen bonds varies substantially with protein identity but is insensitive to KCl concentration

Fig. 5 shows that all halophilic proteins in this study accept significantly more hydrogen bonds from water, per unit area, than their nonhalophilic counterparts. The ferredoxin proteins, in particular, have the highest surface density of water-protein hydrogen bonds than any other protein in their halophilic or mesophilic cohort. In Fig. S1, we show that the number of water-protein hydrogen bonds for each amino acid type (acidic, basic, polar, and apolar) is only weakly sensitive to the identity of the protein and is highest for acidic amino acids. The simulation results are consistent with NMR experiments that demonstrate that the number of water molecules that remain unfrozen at *T* ≪ 0 °C in homopolypeptide solutions—i.e., water molecules perturbed by the homopolypeptides—is substantially higher for the acidic amino acids (71). The variability of the surface density of water-protein hydrogen bonds with protein identity thus largely originates from the different content in acidic amino acids of each protein.

This fact has been used to propose that excess acidic amino acids in halophilic proteins is indispensable to compete with the electrolyte in solution for the available water, thus ensuring that the proteins remain hydrated at high salt concentrations (1,22,24). That possibility, however, is not supported by the results in Fig. 5 and Fig. S1: the surface density of water-protein hydrogen bonds and the number of water-protein hydrogen bonds formed by acidic amino acids are almost identical at *b*_KCl_ = 0.15 mol/kg and at *b*_KCl_ = 2 mol/kg.

But why does K^+^ in solution not compete with the protein for available water at high KCl concentration? To answer this question, we calculate the proximal (also called the perpendicular (72)) number density, *ρ*(*r*), of solvent species at distance *r* to the protein surface:

(14) $$\rho_{X}\left(r\right)=\frac{\bar{n_{X}\left(r\right)}}{dr\;\bar{SASA\left(r\right)}}$$

$\bar{n_{X}\left(r\right)}$ is the time-averaged number of solvent species *X* within a shell of thickness *dr* = 0.05 Å and at distance *r* of the protein surface. This quantity is calculated by determining the distance between each *X* and the nearest nonhydrogen protein atom, as implemented in the rdf function in Gromacs 2018; the position of the oxygen atom is used in the calculation of the number density of water. $\bar{SASA\left(r\right)}$ is the time-averaged protein solvent-accessible surface area, calculated using tcl scripts implemented in VMD; each point of the surface is at distance *r* from the nearest nonhydrogen protein atom.

#### Concentration of water near proteins is highest around halophilic proteins but is insensitive to KCl concentration

**Figure 6:**
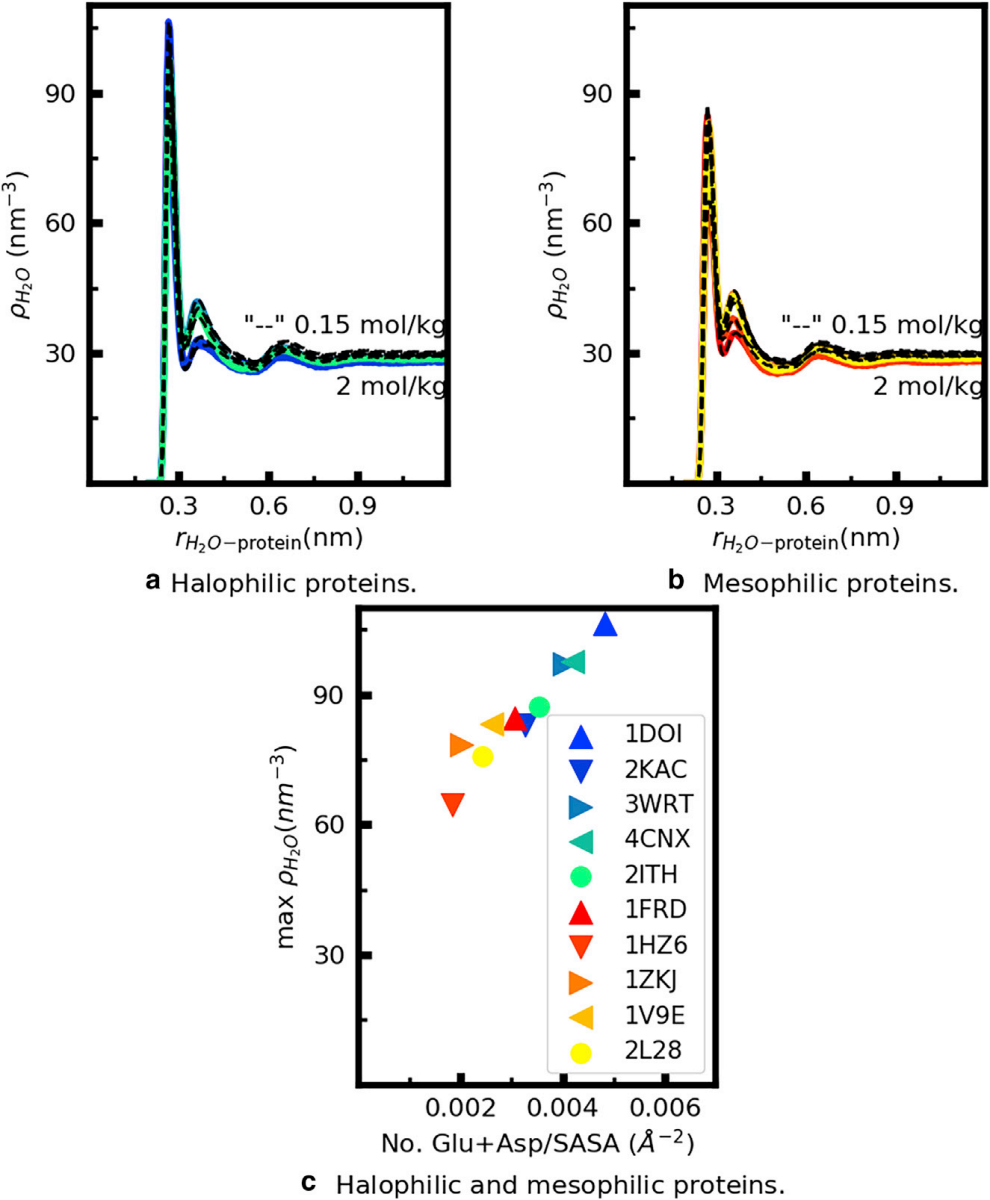
(*a* and *b*) Proximal number density of water molecules as a function of the distance to the surface of the indicated proteins, simulated at *b*_KCl_ = 2 mol/kg (*color*) and *b*_KCl_ = 0.15 mol/kg (*dashed black lines*). (*c*) Height of the first peak of the number density curves for *b*_KCl_ = 2 mol/kg, shown in the other panels, as a function of the surface density of acidic amino acids. Each color corresponds to a protein, identified by its pdb ID in the legend of the bottom panel. To see this figure in color, go online.

In Fig. 6, we show the proximal water number density around the halophilic (Fig. 6 *a*) and mesophilic (Fig. 6 *b*) proteins. The colored curves are obtained at *b*_KCl_ = 2 mol/kg; for *r* < 0.45 nm, overlapping each colored curve is a black, dashed one, obtained from simulations of the same protein simulated at *b*_KCl_ = 0.15 mol/kg. The height of the peaks of the proximal water number density is sensitive to protein identity, and the peaks are on average higher around the halophilic proteins. Irrespective of the halophilic or mesophilic character of each protein, however, the concentration of water in its hydration layer is insensitive to the KCl concentration in the bulk. Moreover, for all proteins and at both KCl concentrations, the water content for *r* < 0.9 nm exceeds the concentration of water in the bulk. These results indicate that proteins do not compete with ions in solution for available water and that mesophilic proteins, despite their lower content in acidic amino acids, are able to retain their hydration layer even at high KCl concentrations.

#### Halophilic and mesophilic proteins perturb water structure to the same length scale

The curves in Fig. 6 retain the same qualitative features despite the different protein sizes and charges. This similarity does not support the hypothesis proposed by several authors (17,19) that the hydration of halophilic proteins is qualitatively different from that of mesophilic ones and that halophilic proteins perturb water structure to particularly large length scales (17). Instead, it is in line with reported calculations of proximal RDFs of water around different proteins, from molecular dynamics simulations and from pdb structures, that indicate that this function has a universal character (72,73). According to this view, differences between the proximal rdfs of different proteins reflect different protein shapes and amino acid composition (72,73). Fig. 6 *c* shows that for our set of proteins, there is a strong correlation between the first maximum of $\rho_{{\text{H}}_{2}\text{O}}\left(r\right)$ and the surface density of acidic amino acids, i.e., between protein hydration and its content in acidic amino acids. Our results suggest that proximal rdfs may be confidently used to aid x-ray refinements of halophilic proteins, using models such as that in (72).

#### Potassium integrates the first hydration shell of both halophilic and mesophilic proteins in a charge-density-dependent manner

**Figure 7:**
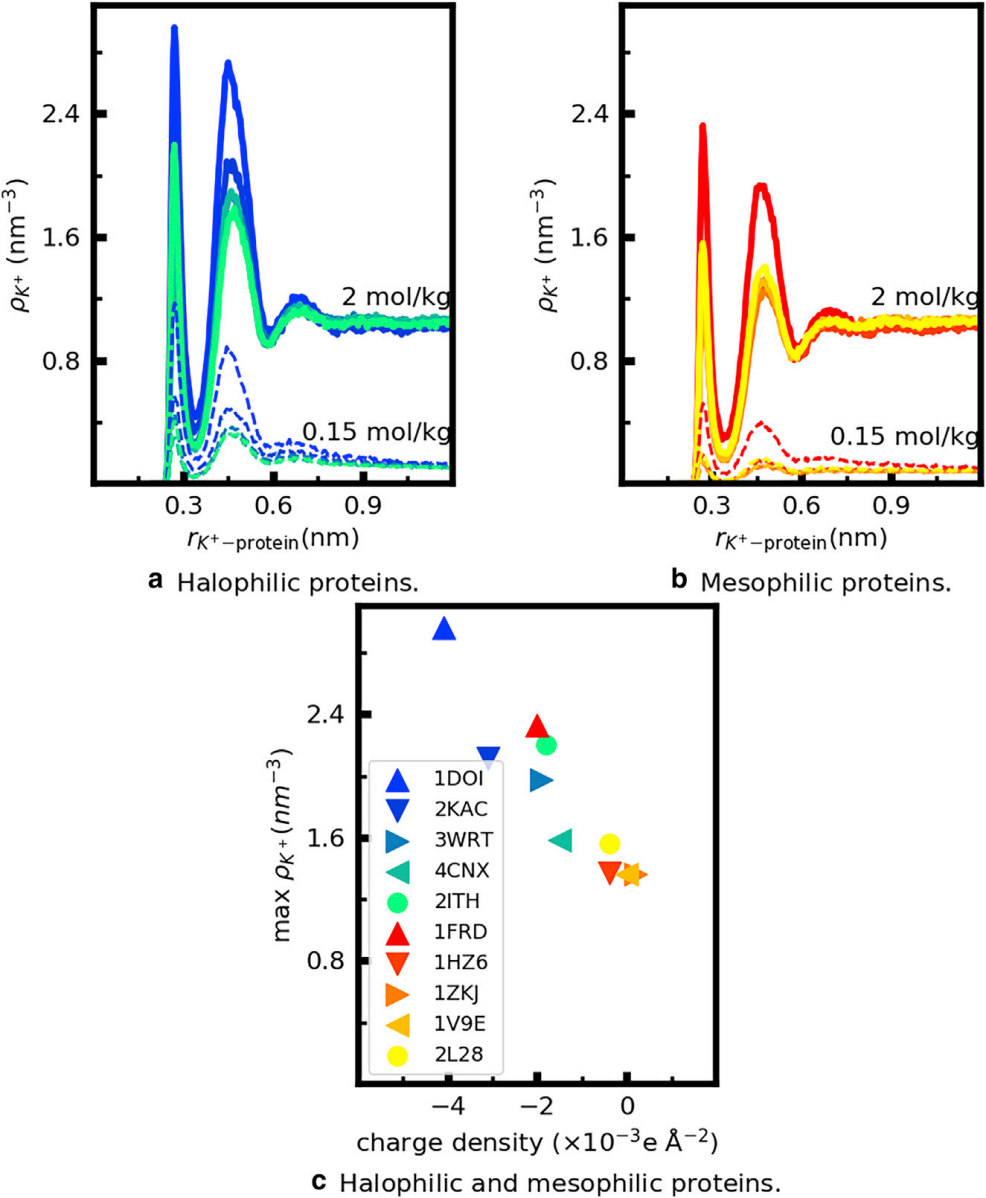
(*a* and *b*) Proximal number density of K^+^ as a function of the distance to the surface of the indicated proteins in solutions with the indicated molality of KCl. (*c*) Height of the first peak of the number density curves for *b*_KCl_ = 2 mol/kg shown in the other panels as a function of the protein charge density. Each color corresponds to a protein, identified by its pdb ID in the legend of the bottom panel. To see this figure in color, go online.

Fig. 7, *a* and *b* shows the proximal number density of K^+^, $\rho_{K^{+}}\left(r\right)$, around each protein. The distributions clarify that K^+^ accumulates in the vicinity (*r* < 0.9 nm) of each protein to concentrations between two and six times higher than in the bulk, depending on the protein identity and the bulk concentration of KCl. The position of the first peak of $\rho_{K^{+}}\left(r\right)$ coincides with that of $\rho_{{\text{H}}_{2}\text{O}}\left(r\right)$ at both low and high *c*_KCl_. Nevertheless, K^+^ cannot measurably displace water from the first hydration shell of the protein because it does not accumulate near the protein in sufficiently large amounts: the height of the peaks of $\rho_{K^{+}}\left(r\right)$ is always below 3 ions/nm^3^; in contrast, the maximal value of $\rho_{{\text{H}}_{2}\text{O}}\left(r\right)$ is ∼105 molecules/nm^3^. Rather than displacing water, K^+^ integrates its own first hydration shell with that of the protein.

Fig. 7, *a* and *b* also shows that the density of K^*+*^ in the vicinity of the protein depends strongly on protein identity and is highest for the halophilic proteins. This origin of dependence is clarified in Fig. 7 *c*, which shows that the height of the first peak of $\rho_{K^{+}}\left(r\right)$ strongly correlates with the charge density of the protein. In other words, the total content of K^+^ in the solvation shell of proteins correlates both with protein net charge and surface area. Halophilic proteins are, on average, larger and have a higher net negative charge than mesophilic ones and, therefore, a larger amount of K^+^ in their solvation layers, as quantified by the cumulative number of potassium ions in the vicinity of each protein shown in Fig. S2.

### Dynamics of the protein solvation shell

We evaluate the impact of salt concentration and protein identity on solvent translational dynamics by calculating the *MSD*(*τ*) of water or of K^+^ according to the following:

(15) $$MSD\left(\tau \right)=\left\langle {\left(\vec{x}\left(t+\tau \right)-\vec{x}\left(t\right)\right)}^{2}\right\rangle$$

$\vec{x}\left(t\right)$ is the position of each species at time *t* and the bracket indicates both a time and an ensemble average over the relevant species population. We calculate the MSD for water or K^+^ belonging to the first solvation layer at t=0 because if halophilic proteins indeed substantially slow down water dynamics, this effect should be strongest for these subpopulations. We are interested in the short-time dynamics of each species because this dynamics should reflect the strongest impact of their initial position in the hydration shell and thus differ the most from the dynamics of the same species averaged over all its elements in the simulation box. For this reason, it is sufficient to calculate the MSD using coordinates wrapped back into the main simulation cell.

In Figs. S3 and S4, we show the MSD of water and of K^+^ in the first hydration layer around the halophilic and mesophilic ferredoxin proteins; the results for the other proteins (data not shown) are qualitatively similar. As expected under these conditions, the curves saturate at long times, which is evidence of confinement to the main simulation cell. To facilitate comparisons between different proteins and simulation conditions, we calculate the diffusion coefficient, *D*, as

(16) MSD(τ)=6Dτ+C,

by fitting each *MSD*(*τ*) curve in the interval *τ* = [20, 30] ps. The constant *C* is added to account for nondiffusive movement at short times. This time interval is chosen because the dynamics of all species is already diffusive beyond 20 ps, but the impact of confinement by the finite simulation box remains marginal much beyond 30 ps (see example in Fig. S3). To facilitate comparisons, the diffusion coefficient averaged over all K^+^ or water molecules in the simulation box (calculated using unwrapped coordinates) is estimated from fits using the same time interval.

#### Halophilic and mesophilic proteins have solvation layers with identical translational dynamics at both low and high KCl concentrations

**Figure 8:**
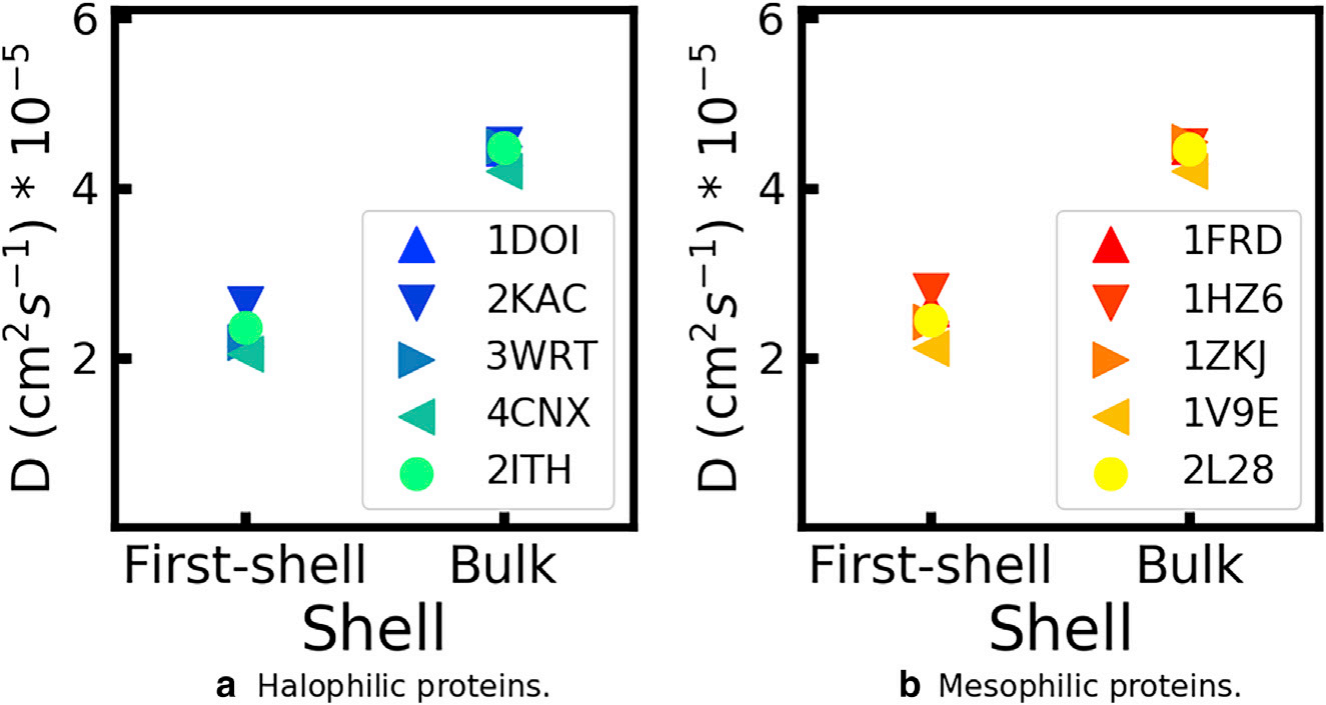
Diffusion coefficients of water around the indicated proteins, simulated in *b*_KCl_ = 2 mol/kg. The first shell consists of water molecules that, at *t* = 0, belong to the first hydration shell of the proteins; bulk consists of all water molecules in the same simulation. To see this figure in color, go online.

In Fig. 8, we compare the diffusion coefficients of water molecules that initially belong to the first hydration layer of each protein with the diffusion coefficient of all water molecules in each simulation, at *b*_KCl_ = 2 mol/kg; results for *b*_KCl_ = 0.15 mol/kg are shown in Fig. S5. The TIP3P water model is known to predict translational and rotational dynamics, which are approximately twice faster than the reference experimental values (74). Accordingly, the absolute values of $D_{{\text{H}}_{2}\text{O}}$ reported in Fig. 8 are likely overestimated; only their relative magnitude is meaningful. The translational dynamics in the first hydration layer is indeed lower than that of water in the bulk but only by a factor of 1/2 at most. Moreover, the translational dynamics of water in the first hydration layer of the mesophilic proteins is barely faster that of the nonhalophilic proteins. Our results are not consistent with the possibility that water near halophilic proteins has dramatically slower dynamics than around nonhalphilic ones.

**Figure 9:**
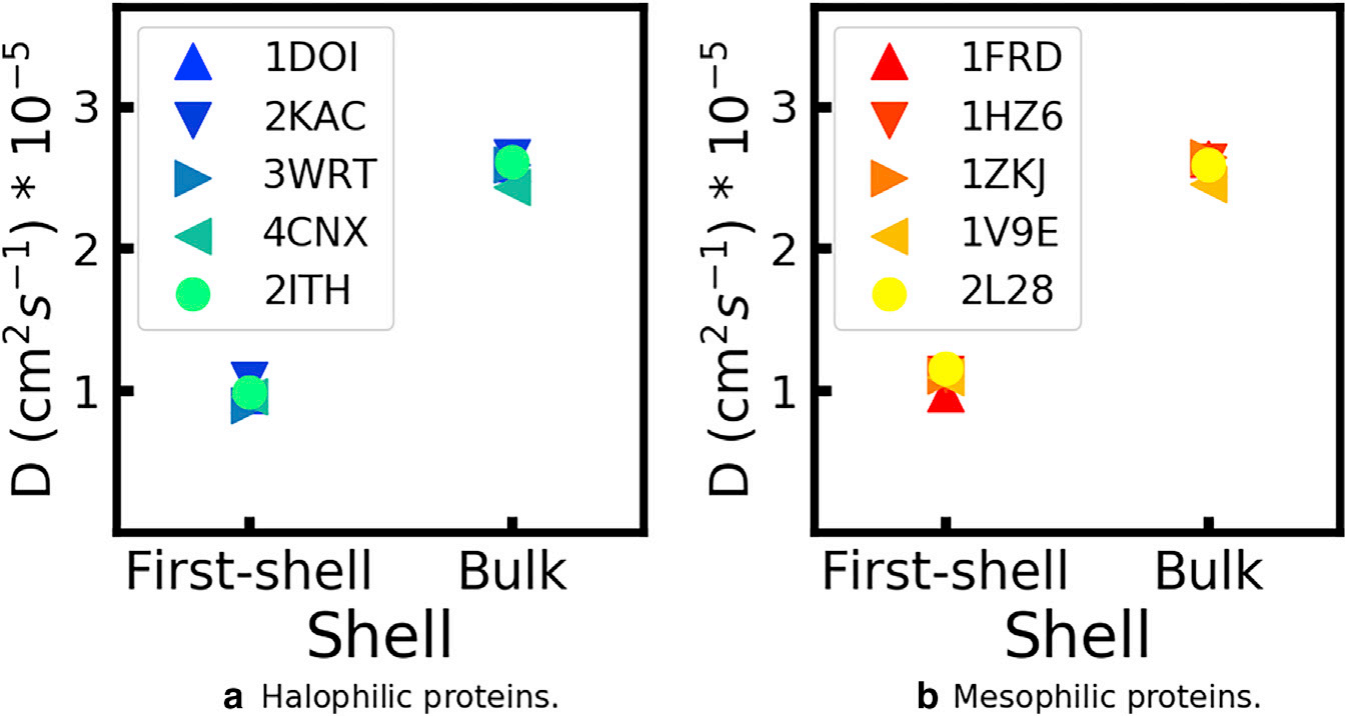
Diffusion coefficients of potassium ions around the indicated proteins, simulated in *b*_KCl_ = 2 mol/kg. The first shell consists of potassium ions that, at *t* = 0, belong to the first solvation shell of the proteins; bulk consists of all potassium ions in the same simulation. To see this figure in color, go online.

Fig. 9 compares the self-diffusion coefficients of potassium ions initially in the first solvation layer of the protein with those averaged over all K^+^ in the simulation box, again at the highest salt concentration (see Fig. S6 for the results at *b*_KCl_ = 0.15 mol/kg). Similar to the trends observed for water dynamics, the translational dynamics of K^+^ in the first solvation layer is lower than dynamics in the bulk but only by a factor of 1/3. Moreover, the diffusion coefficients of this subpopulation are very similar for the halophilic and mesophilic proteins. Even though halophilic and mesophilic proteins differ substantially in surface charge density and these differences lead to considerable differences in the number of K^+^ in the solvation layer of each protein (Fig. S2), they do not impact the translational dynamics of the ions near the protein.

## Discussion

Both the solvent-only and the ion-solvent stabilization models propose that excess acidic amino acids are necessary in halophilic proteins to maintain their hydration at high salt concentrations. Our results do not support this scenario. Halophilic proteins do accept more hydrogen bonds from water (Fig. 5) and have larger concentration of water in their hydration shells (Fig. 6) because of their higher content in acidic amino acids (Fig. 6 *c*; Fig. S1; Table S3). These results are consistent with sedimentation measurements (18,19), which indicate that halophilic proteins bind larger amounts of water than mesophilic ones. That fact does not imply that excess acidic amino acids are necessary to maintain protein hydration at high KCl concentration, however. Our study demonstrates that all mesophilic and halophilic proteins investigated here remain equally well hydrated at both low (*b*_KCl_ = 0.15 mol/kg) and high (*b*_KCl_ = 2 mol/kg) KCl concentration, as measured by the number of hydrogen bonds that they accept from water (Fig. 5) and the water concentration at a distance below 4.5 Å from the protein surface (Fig. 6). These results imply that the proteins simulated here do not compete with ions in solution for available water even at concentrations as high as *b*_KCl_ = 2 mol/kg. The force fields used here for carboxylates and K^+^ ((36) and this work) were developed to reproduce experimentally determined hydration free energies, lending confidence to our observations. The proteins selected for this study have diverse sizes, net charges, and surface charge density and include both typical examples of halophilic and mesophilic proteins as well as extreme examples (the ferredoxin proteins) of each category (Table S3). The conclusion that protein hydration is concentration independent for *b*_KCl_ ≤ 2 mol/kg and that proteins do not compete with KCl for available water thus may apply to all proteins.

Moreover, we suggest that this conclusion may hold also for aqueous NaCl solutions because the water activity of NaCl and KCl solutions is very similar (Fig. S8, based on (75)). The limited experimental data comparing protein hydration in NaCl vs. KCl solutions is consistent with this possibility: neutron scattering and ultracentrifugation studies show that halophilic malate dehydrogenase binds equally large amounts of water when immersed in KCl or in NaCl solutions (18). We note, however, that Na^*+*^ is expected to form more contact ion pairs with carboxylates than K^*+*^ (compare the acetate..K^+^ and acetate..Na^+^ RDFs shown in Fig. S7, and also prior work by others (65,66,68)). Differences in the stability and dynamics of halophilic proteins in NaCl and KCl solutions (76) likely reflect the different interactions between the cations in solution and the acidic amino acids in the protein. It is of interest to comparatively characterize protein solvation shells in KCl vs. NaCl solutions using nonperturbative methods such as solvation-shell spectroscopy (77) and to understand how their differences influence the conformational stability and the functionality of enzymes. This understanding is critical to rationally develop proteins for use in biotechnological devices (e.g., to produce molecular hydrogen) that function in brackish or sea water, thus reducing pressure on our planet’s freshwater resources.

The water activity decreases from 1 to 0.8 as the concentration of KCl increases to *b*_KCl_ ≤ 4 mol/kg (Fig. S8). Low water activity also occurs in solvent mixtures composed of water and organic liquids. At first sight, halophilic proteins offer clues to understand which sequence-structure features enable the ability to function at low water activity arising from the presence of organic solvents; in fact, some halophilic proteins remain functional under these conditions (78,79). A closer look into the limited available data, however, suggests that protein hydration may respond very differently to the two environments and that further studies are necessary before drawing analogies between the proteins at high salt concentration and proteins in mixtures of water with organic solvents. Our results indicate that protein hydration is insensitive to KCl concentrations up to *b*_KCl_ = 2 mol/kg, i.e., protein hydration levels are unaffected by changes in water activity between 1 and 0.9. In contrast, the fraction of water bound to proteins decreases from 60% to 40% (weight percentage of bound water in the protein+bound water system isolated by centrifugation) in solutions of water with immiscible organic solvents (80,81) as the water activity decreases from 1 to 0.9. It is at present unclear whether the different dependence between protein hydration and water activity is real or whether it reflects a bias imposed by the different observables being compared. There is a clear need for nonperturbative experiments to comparatively characterize the hydration shells of proteins in aqueous electrolyte media and in aqueous media containing organic solvents to assert the extent to which halophilic proteins may be useful starting points to design enzymes and catalysts that function in low water activity environments that occur, e.g., when producing ethanol in large quantities or when catalyzing reactions in the presence of organic solvents.

The ion-solvent stabilization hypothesis proposes that cooperative ion-solvent-protein networks arise in halophilic proteins because of their tertiary and quaternary structure (18,19); cooperative interactions would not exist in the unfolded or nonoligomerized state of the protein because in that state, the acidic amino acids are further apart or in unfavorable orientation. These networks have been proposed to explain experimental observations (based on analysis of centrifugates), showing that halophilic proteins bind more cations than mesophilic ones (18,19). Our simulation results agree with the experiment: halophilic proteins indeed have larger amounts of K^+^ in their solvation shell than mesophilic proteins (Fig. 7). The fact that the maximum of the number density of K^+^ around the proteins correlates linearly with the charge density of the protein (Fig. 7 *c*) suggests that cation binding has a predominantly electrostatic origin. We are currently investigating whether protein-ion-solvent interactions indeed have a cooperative component.

Neutron scattering experiments have suggested that the cytoplasm of halophilic organisms has a fraction of water with translational diffusion ∼250 times slower than that of water in the bulk; in contrast, that slow component was not observed in mesophilic organisms (21). A fraction of water with extremely slow dynamics has been proposed to arise in the context of the ion-solvent stabilization model: the cooperative ion-water-protein networks would strongly reduce the mobility of water in the first hydration layer of the protein (17). Our results do not support this view: the translational dynamics of water and of K^+^ near halophilic and mesophilic proteins is indistinguishable, and only two to three times slower than the dynamics of the same species in the bulk (Figs. 8 and 9). Our simulations are consistent with ^17^O magnetic relaxation measurements of halophilic and mesophilic versions of protein L (one of the protein pairs simulated here), which show that they have similar hydration dynamics (23). This result does not imply that cooperative ion-solvent-protein interactions are necessarily absent at high KCl concentrations. Cooperative interactions may well exist, but our results indicate that they should not result on unusually slow solvent dynamics, i.e., they do not come at the expense of a high entropic price.

## Conclusions

Many halophilic organisms contain molar concentrations of KCl in their cytoplasm, necessary to balance the large osmotic pressure induced by equally high external concentrations of NaCl (5). The cytoplasmic proteins of these organisms are substantially richer in acidic amino acids than mesophilic ones (1). Because acidic amino acids can bind substantially larger amounts of water than any other natural amino acid (71), it has been proposed that halophilic proteins require acidic amino acids to remain hydrated in their low water activity environment (1,24). This work does not support this possibility. Our simulations of five halophilic proteins and five mesophilic counterparts indicate that halophilic proteins indeed contain larger amounts of water in their hydration shells than mesophilic ones and that their larger hydration level correlates with their high content in acidic amino acids. However, all proteins remained equally hydrated at low (*b*_KCl_ ≤ 0.15 mol/kg) and high (*b*_KCl_ ≤ 2 mol/kg) KCl concentrations, demonstrating that a higher content in acidic amino acids is not necessary to remain hydrated at high KCl concentrations. We note, however, that to understand the connection between acidic amino acids, solvation, and protein stability, it is also necessary to investigate the unfolded state of proteins; it is the difference in protein hydration and protein-salt interactions between the folded state and the unfolded ensemble that matters (82,83). We will focus on this point in future studies.

Cooperative interactions between acidic amino acids, water and cations have been proposed to exist in the folded structure of halophilic proteins because their high content in acidic amino acids would enable specific, favorable ion-water-carboxylate configurations to form (18,19,22). According to this view, these interactions would stabilize the folded protein configuration, they would be necessary to maintain the protein hydrated at high KCl concentration (18,19,22), and would manifest themselves in a fraction of water with very slow translational dynamics (21). Our simulations show that halophilic proteins have a higher concentration of K^+^ in their solvation shells than mesophilic ones, consistent with experiment (18,19). The concentration of K^+^ in the protein solvation shell linearly correlates with the protein net charge/SASA ratio. Halophilic proteins are more negative than mesophilic ones, hence the higher concentration of K^+^ in their hydration shells. Despite this high concentration, the solvation shell of halophilic proteins remains as labile as that of mesophilic ones, and we find no evidence of water or ions with unusually slow translational dynamics around halophilic proteins. The absence of slow dynamics, we point out, does not imply that cooperativity is impossible; it suggests, however, that if present, it does not unusually slow down water dynamics. If a stabilizing interaction between acidic amino acids (mediated by K^+^) indeed exists and is more prevalent in folded rather than unfolded protein conformations, it might be one of the driving forces behind the abundance of acidic amino acids in halophilic proteins. Conversely, if the interactions between acidic amino acids are predominantly destabilizing, they might be necessary to retain protein flexibility (17) or simply to prevent aggregation in a media that greatly enhances the hydrophobic effect (15). We are currently investigating whether water-cation-carboxylate interactions in halophilic proteins have a cooperative nature and the impact of the content in acidic amino acids on protein conformational dynamics and protein-protein interaction.

## Author contributions

A.V.V. designed and guided the research. H.G.D. performed the research. Both authors wrote the manuscript.

## Supporting material

Document S1. Supporting materials and methods, Figs. S1–S8, Tables S1–S3

Data S1. Amber ff14SB, dat, and lib files modified to include the optimized parameters

Document S2. Article plus supporting material
