# Supplementary material for: Proteins maintain hydration at high [KCl] concentration regardless of content in acidic amino acids
Source: Biophys J. 2021 Jun 2;120(13):2746–62. doi: 10.1016/j.bpj.2021.05.015 (PMC8390907; doi:10.1016/j.bpj.2021.05.015)
Supplement: Document S1. Supporting materials and methods, Figs. S1–S8, Tables S1–S3 [file mmc1.pdf]

Biophysical Journal, Volume *120*

Supplemental information

Proteins maintain hydration at high [KCl] concentration regardless of content in acidic amino acids

Hosein Geraili Daronkola and Ana Vila Verde

Supporting information for
Proteins maintain hydration at high [KCl] concentration regardless of content in acidic amino acids

Hosein Geraili[a], Ana Vila Verde*[a,b]

1 Reference crystallographic distances between K^+ and carboxylate oxygens used for parameterization

Table 1 Distance between K^+ and the indicated carboxylate oxygens of acidic amino acids in an halophilic ferredoxin (pdb ID 1DOI), from crystallography and from simulation. Related to Fig. 4 of the main text.

		$f_{R_{min,K^+O}}$					
		1	1.04	1.06	1.08	1.09	1.1
Prot. site[a]	$r^b_{cryst.}$ (Å)	r^c_{sim} (Å)					
12ASP_OD1	3.01	2.678	2.833	2.859	2.942	2.993	2.846
81ASP_OD2	2.97	2.671	2.832	2.848	2.894	2.822	2.75
107ASP_OD1	3.08	2.68	2.834	2.893	2.946	2.985	2.976
109ASP_OD1	3.12	2.688	2.853	2.888	2.887	2.834	2.813
110GLU_OE2	2.8	2.674	2.807	2.911	2.836	2.808	2.832

(a) The protein site is identified by the residue number, residue name and oxygen name.
(b) $r_{cryst.}$: distances reported in ref. 1 for the 1DOI crystal structure.
(c) $r_{sim.}$: position of the first peak of the RDFs calculated using molecular dynamics simulations of the same protein at room temperature, solvated in 1 mol/dm^3 KCl and with constrained backbone, using the indicated parameter values for the interaction between K^+ and the oxygen carboxylates.

2 Properties of the simulated halophilic and mesophilic proteins

In SI Table 2 we report the structural and sequence similarity of the proteins studied. The RMSD values in Table 2 are calculated for C^{α} atoms of the backbone of the amino acids based on which the two proteins are aligned using the program STAMP (Structural Alignment of Multiple Proteins)[2].The Q_H value is a metric for structural homology which calculates the similarity of two structures by considering their amino acid similarity and adding a term for any gap in the alignment[3]. Proteins showing a value of $Q_H > 0.6$ are considered to have good structural conservation. In SI Table 3 we show the average amino acid composition and charge for all proteins.

Table 2 Halophilic-mesophilic protein pairs.

Halophiles		Mesophiles			Comparison		
PDB[a]	Organism[b]	PDB[a]	Organism[b]	name[c]	RMSD (Å)[d]	Sequence identity (%)[e]	Q_H[f]
1DOI	Haloarcula marismortui	1FRD	Anabaena 7120	Ferredoxin	1.54	22	0.636
2KAC	Peptostreptococcus magnus	1HZ6	Peptostreptococcus magnus	Protein L	0.95	75	0.877
3WRT	Chromohalobacter sp. 560	1ZKJ	Enterobacter aerogenes	Beta-lactamase	2.23	44	0.680
4CNX	Bos taurus	1V9E	mammalian enzyme	Carbonic anhydrase	1.30	90	0.857
2ITH	Haloferax volcanii	2L28	Lactobacillus casei	Dihydrofolate reductase	2.41	19	0.595

(a) Protein Data Bank code
(b) Source organisms
(c) Protein name as reported in the Protein Data Bank
(d) Root Mean Square Deviation between each pair of halophilic-mesophilic proteins, calculated using VMD.[4]
(e) Sequence identity between each protein pair, calculated using the MultiSeq plugin[5] in VMD, based on the algorithm described in ref.[5].
(f) A metric of structural homology.

Table 3 Length (n_{aa}), number of acidic (n_{acidic}) and basic (n_{basic}) amino acids and charge of the simulated proteins. The protein charge is defined by the difference between acidic and basic amino acids, and by the charge of metal ligands if present.

Halophiles					Mesophiles				
pdb	n_{aa}	n_{acidic}	n_{basic}	charge (e)	pdb	n_{aa}	n_{acidic}	n_{basic}	charge (e)
1DOI	128	34	7	-29	1FRD	98	18	8	-12
2KAC	64	16	1	-15	1HZ6	72	10	8	-2
3RWT	367	57	30	-27	1ZKJ	359	29	32	+3
4CNX	259	48	29	-17	1V9E	256	30	28	0
2ITH	162	30	15	-15	2L28	162	22	18	-4

3 Water-protein hydrogen bonds per residue type

Fig. 1 shows the average number of hydrogen bonds donated by water molecules to the protein, averaged separately for different types of residues. Acidic residues accept far more hydrogen bonds than any other residue type. The number of hydrogen bonds accepted by any given residue depends weakly (if at all) on protein identity and on the concentration of KCl in the bulk.

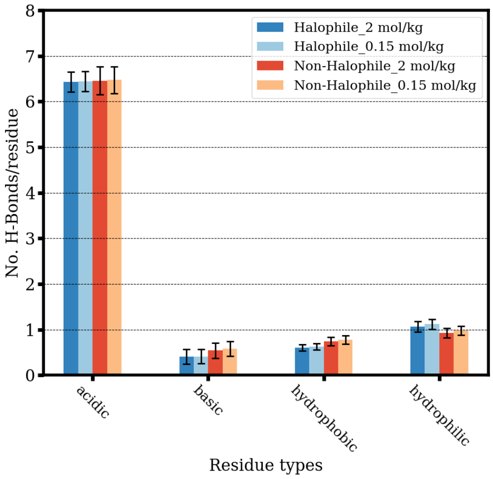

(a) 1DOI_1FRD protein pair

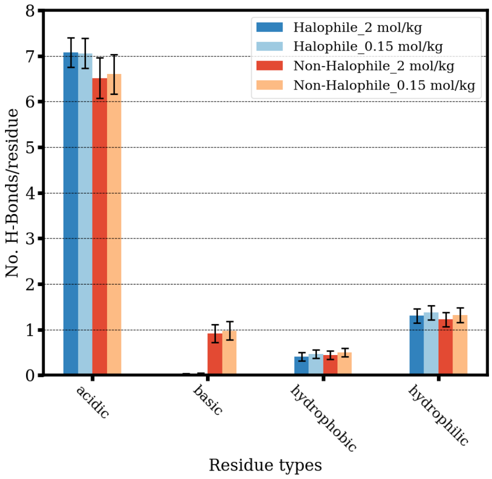

(b) 2KAC_1HZ6 protein pair

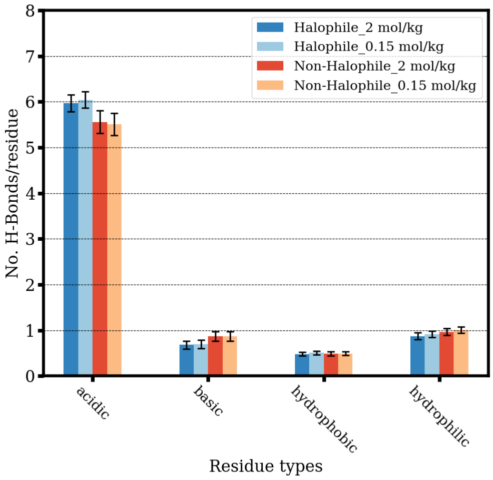

(c) 4CNX_1V9E protein pair

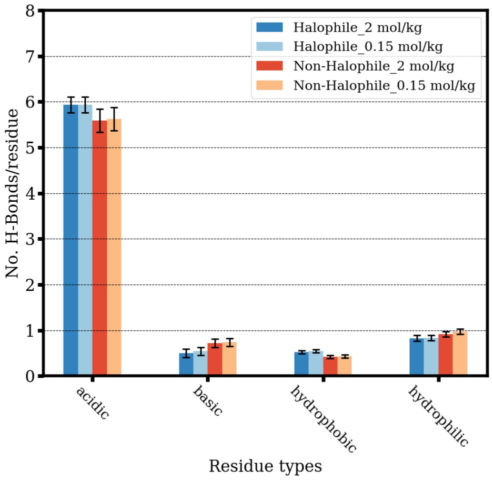

(d) 3WRT_1ZKJ protein pair

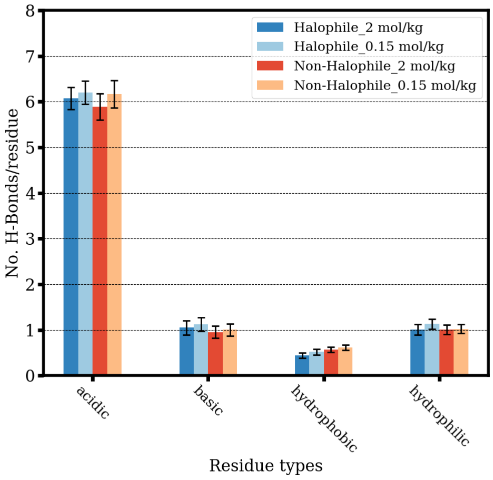

(e) 2ITH_2L28 protein pair

Fig. 1 Number of water-protein hydrogen-bonds per residue, averaged over the indicated amino acid types (acidic, basic, hydrophobic, and hydrophilic (=polar, non-charged), for the indicated halophilic-mesophilic proteins, identified by their pdb IDs.

4 Cumulative number of potassium ions as a function of distance to the protein surface

Fig. 2 shows the number of potassium ions within any given distance to the protein surface. The largest differences between halophilic proteins and their mesophilic counterpart are seen for the ferredoxin (Fig. 2a) and the beta-lactamase (Fig. 2d). These large differences reflect not only their different amino acid composition, but also their different size (in the case of ferredoxin) and shape (beta-lactamase)

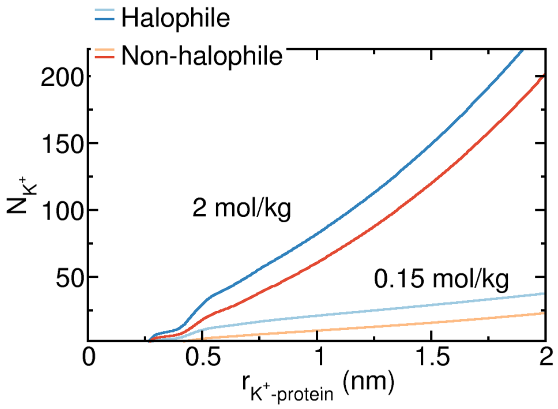

(a) 1DOI_1FRD protein pair

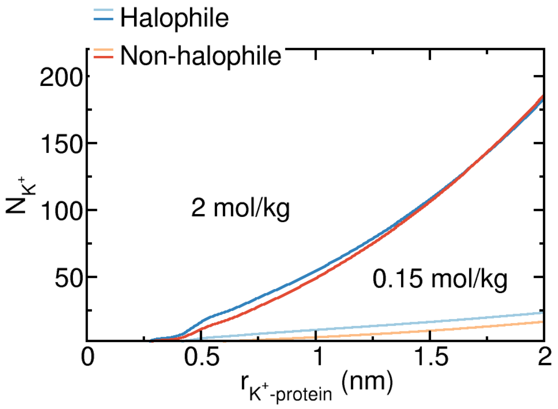

(b) 2KAC_1HZ6 protein pair

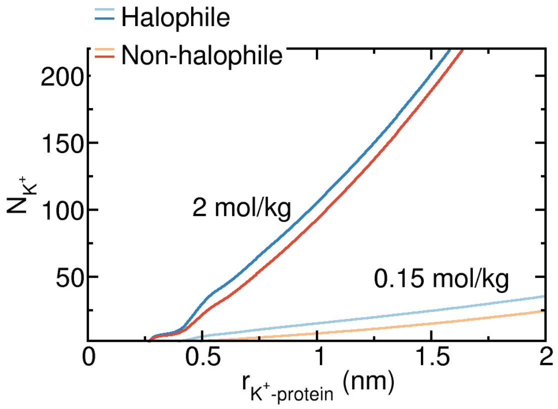

(c) 4CNX_1V9E protein pair

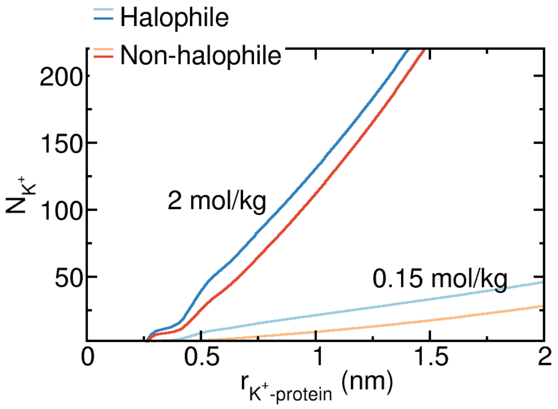

(d) 3WRT_1ZKJ protein pair

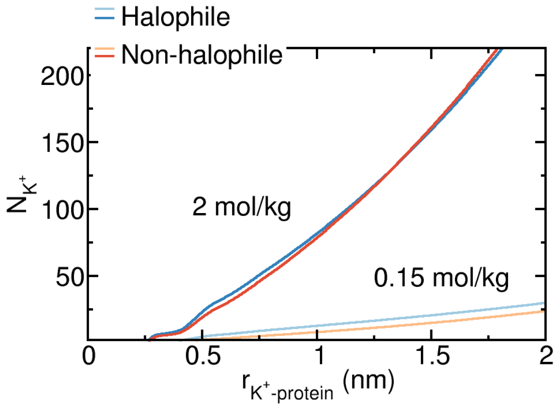

(e) 2ITH_2L28 protein pair

Fig. 2 Cumulative number of potassium ions as a function of the distance to the heavy atoms defining the protein surface, from simulations at the indicated concentration of KCl.

5 Mean square displacement of water and of K^+

SI Figs. 3 and 4 show the MSD of water or K^+ ions belonging to the first hydration shell of each ferredoxin protein, simulated at high and low KCl concentration, calculated as described in the main text. The MSD of water and of K^+ calculated for the other proteins (results not shown) are qualitatively similar to these. For $t < 10$ ps, both water and K^+ are clearly in the subdiffusive regime. The diffusive regime is only seen for $t = [10, 50]$ ps; beyond this time, the particles move supradiffusively for a short time interval. For $t > 1$ ns the MSD saturates, reflecting the fact that this MSD is calculated using coordinates wrapped back to the main simulation cell.

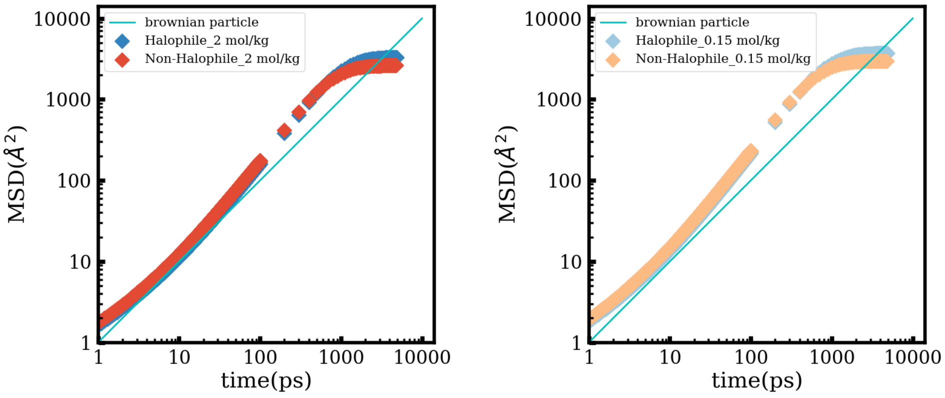

(a) b_{KCl} = 2 mol/kg.

(b) b_{KCl} = 0.15 mol/kg.

Fig. 3 MSD of the subpopulation of water molecules that belong to the first hydration shell of the halophilic or non-halophilic ferredoxin at $t = 0$, for the indicated salt concentration. The light blue line illustrates the diffusive limit of a Brownian particle with $D = 1/6$ Å^2/ps.

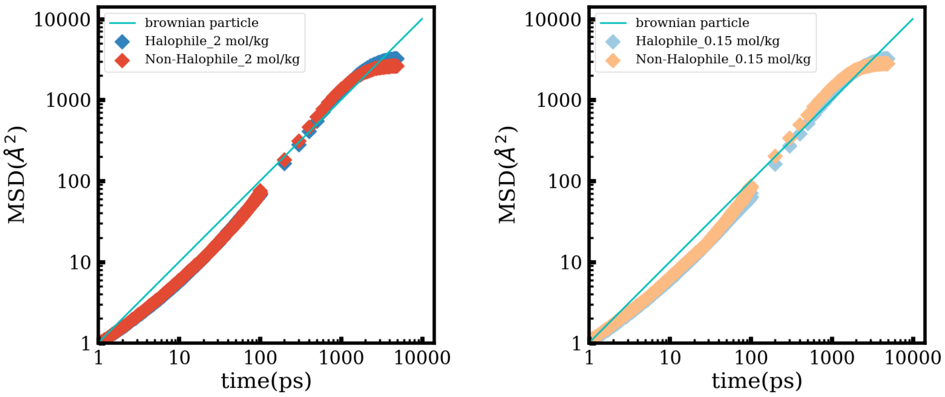

(a) b_{KCl} = 2 mol/kg.

(b) b_{KCl} = 0.15 mol/kg.

Fig. 4 MSD of the subpopulation of K^+ ions that belong to the first hydration shell of the halophilic or non-halophilic ferredoxin at $t = 0$, for the indicated salt concentrations. The light blue line illustrates the diffusive limit of a Brownian particle with $D = 1/6$ Å^2/ps.

In Figs. 5 and 6 we show the diffusion coefficients, D, of water and of potassium for all proteins at b_{KCl} = 0.15 mol/kg. Details of the calculation of D are given in the main text.

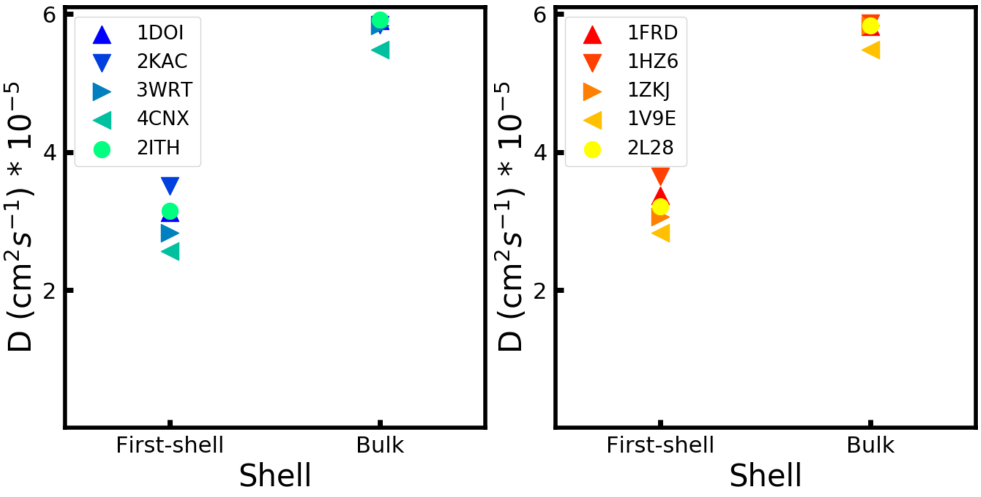

(a) Halophiles **(b)** Non-Halophiles

Fig. 5 Diffusion coefficients of: (First-shell) water molecules that belong to the first hydration shell of the indicated proteins at $t = 0$, simulated at b_{KCl} = 0.15 mol/kg; (Bulk) all water molecules in the same simulation.

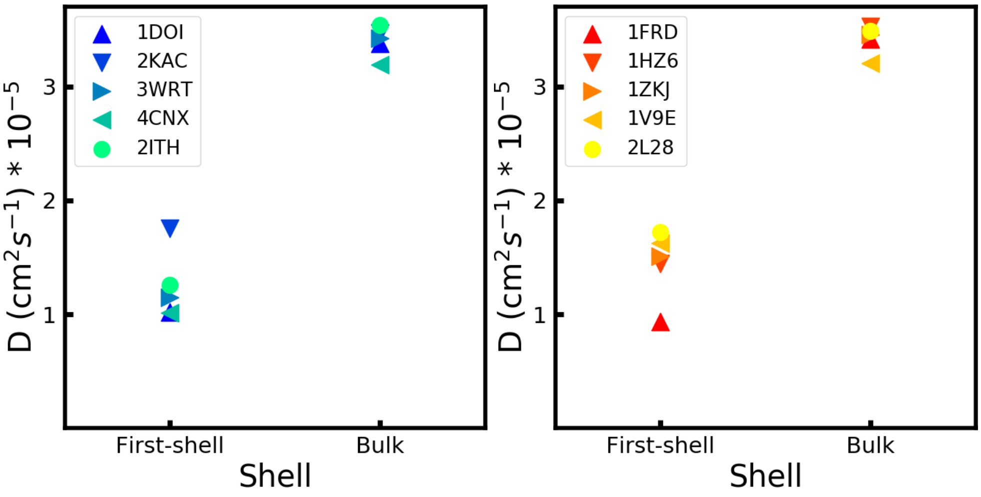

(a) Halophiles **(b)** Non-Halophiles

Fig. 6 Diffusion coefficients of: (First-shell) potassium ions that belong to the first solvation shell of the indicated proteins at $t = 0$, simulated at b_{KCl} = 0.15 mol/kg; (Bulk) all potassium ions in the same simulation.

6 Ion pairing in potassium acetate and sodium acetate solutions

Fig. 7 shows that contact ion pairs (CIP) are more abundant in $NaCH_3COO$ than in KCH_3COO. In both cases, however, the solution is dominated by solvent shared ion pairs (SIP).

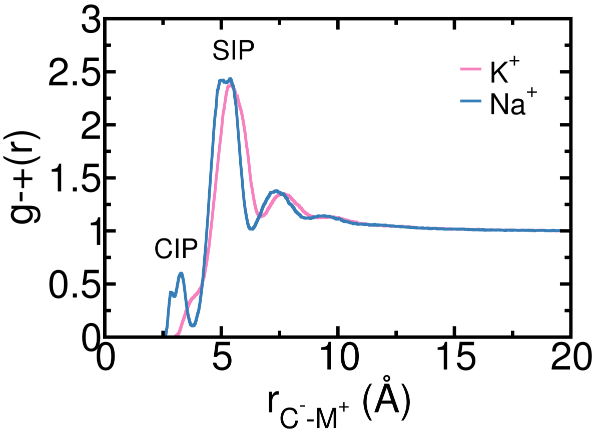

Fig. 7 Radial distribution function between the metal ion (M^+) and the carboxylate carbon (C^-), from simulations of aqueous solutions of $NaCH_3COO$ with molality 0.5 mol/kg, or KCH_3COO at the same concentration. The parameters for the interaction between carboxylate and water and between carboxylate and Na^+ are from ref. 6; the interaction between carboxylate and K^+ is modelled using the optimized parameter shown in Table 1 of the main text. The remaining parameters are from GAFF and TIP3P water.

7 Water activity in NaCl and KCl solutions

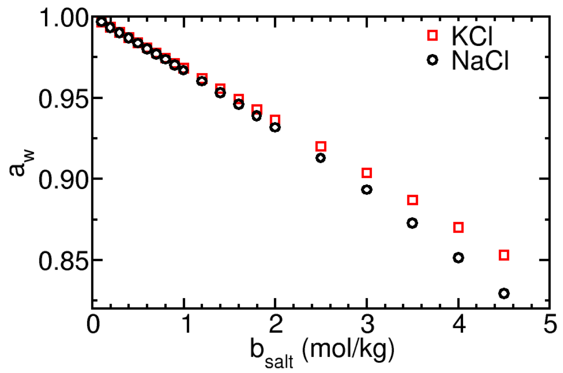

Fig. 8 Experimentally determined water activity (a_w) in solutions of NaCl or KCl with the indicated molality, b. From ref. 7.

References

1 F. Frolow, M. Harel, J. L. Sussman, M. Mevarech and M. Shoham, *Nat. Struct. Mol. Biol.*, 1996, **3**, 452–458.

2 R. B. Russell and G. J. Barton, *Proteins.*, 1992, **14**, 309–323.

3 P. O'Donoghue and Z. Luthey-Schulten, *Microbiol. Mol. Biol. Rev.*, 2003, **67**, 550–573.

4 W. Humphrey, A. Dalke and K. Schulten, *J. Mol. Graph.*, 1996, **14**, 33–38.

5 E. Roberts, J. Eargle, D. Wright and Z. Luthey-Schulten, *BMC Bioinformatics*, 2006, **7**:382.

6 S. Kashefolgheta and A. Vila Verde, *Phys. Chem. Chem. Phys.*, 2017, **19**, 20593–20607.

7 R. A. Robinson and R. H. Stokes, *Trans. Faraday Soc.*, 1949, **45**, 612–624.
